# Supplementary material for: Pulmonary valve tissue engineering strategies in large animal models
Source: PLoS One. 2021 Oct 5;16(10):e0258046. doi: 10.1371/journal.pone.0258046 (PMC8491907; doi:10.1371/journal.pone.0258046)
Supplement: S1 Fig — Follow-up time of each comparison in the meta-analyses showed equal variation in the natural and synthetic scaffolds in valve regurgitation (A) and pressure (B) gradient analyses. (DOCX) [file pone.0258046.s002.docx]

**Fig. S1. Follow-up time of each comparison in the meta-analyses.**

**B. Pressure gradient (mean)**

**A. Moderate/severe valve regurgitation**

**Fig.S1 Follow-up time of each comparison in the meta-analyses**. Follow-up time of each comparison in the meta-analyses showed equal variation in the natural and synthetic scaffolds in valve regurgitation **(A)** and pressure **(B)** gradient analyses.
