# Supplementary material for: Pulmonary valve tissue engineering strategies in large animal models
Source: PLoS One. 2021 Oct 5;16(10):e0258046. doi: 10.1371/journal.pone.0258046 (PMC8491907; doi:10.1371/journal.pone.0258046)
Supplement: S1 Table — (DOCX) [file pone.0258046.s003.docx]

**S1 Table. Syntax OVID Medline and Embase database.**

| **#** | **Searches** | **Database** |
| --- | --- | --- |
| **1** | goats/  or exp sheep/  or exp sus scrofa / or primates / or exp cercopithecinae / or exp papio / or dogs / or (cow or cows or bull or bulls or (calves not (calves adj3 (patient* or pain*))) or goat* or capra or capras or sheep or ovis or ovine or ewe or ewes or aries or lamb or lambs or sus or Suidae or swine* or sow or sows or gilt or gilts or pig or pigs or piglet* or minipig* or primate* or monkey* or ape or apes or baboon* or papio or dog or dogs or bitch or bitches or canine or beagle* or pup or pups).tw,kf . | ***Pubmed (Medline)*** |
|  | Exp goat/ or exp sheep/ or exp pig/ or primate/ or [several primate species?] or exp baboon/ or exp dog/ or (cow or cows or bull or bulls or (calves not (calves adj3 (patient* or pain*))) or goat* or capra or capras or sheep or ovis or ovine or ewe or ewes or aries or lamb or lambs or sus or Suidae or swine* or sow or sows or gilt or gilts or pig or pigs or piglet* or minipig* or primate* or monkey* or ape or apes or baboon* or papio or dog or dogs or bitch or bitches or canine or beagle* or pup or pups).ti,ab,kw,hw. | ***Embase*** |
| **2** | (calf or calves).tw,kf. and (cattle or animal*).mp . | ***Pubmed (Medline)*** |
|  | (calf or calves).ti,ab,kw,hw. and (cattle or animal*).mp. | ***Embase*** |
| **3** | ((porcine or bovine or calf) adj3 model*).tw,kf. | ***Pubmed (Medline)*** |
|  | ((porcine or bovine or calf) adj3 model*).ti,ab,kw,hw. | ***Embase*** |
| **4** | (models, animal/ or disease models, animal/ or animal experimentation/ or laboratory animal science/ or exp animals, laboratory/ or (models, cardiovascular/ and animals/) or (((animal or in vivo) adj3 experiment*) or ((animal* or experimental or preclinical or in-vivo) adj3 (model* or study))).tw,kf.) and (porcine* or bovine or swine or cattle).mp. | ***Pubmed (Medline)*** |
|  | Animal model/ or bovine model/ or canine model/ or caprine model/ or equine model/ or feline model/ or ovine model/ or porcine model/ or exp primate model/ or exp animal experiment/ or laboratory/ or experimental animal/ or exp experimental cat/ or exp experimental cattle/ or experimental dog/ or experimental goat/ or exp experimental horse/ or exp experimental monkey/ or exp experimental pig/ or exp experimental sheep/ or germfree animal/ or spinal animal/ or (((animal or in vivo) adj3 experiment*) or ((animal* or experimental or preclinical or in-vivo) adj3 (model* or study))).ti,ab,kw,hw. | ***Embase*** |
| **5** | (models, animal/ or disease models, animal/ or animal experimentation/ or laboratory animal science/ or exp animals, laboratory/) not (exp rodentia / or rabbits/  or (mouse or mice or rat or rats or rabbit or rodent*).ti.) | ***Pubmed (Medline)*** |
|  | (Animal model/ or exp animal experiment/ or laboratory/ or experimental animal/) not (exp rodent/ or exp rabbits/ or (mouse or mice or rat or rats or rabbit or rodent*).ti.) | ***Embase*** |
| **6** | large animal*.tw,kf. | ***Pubmed (Medline)*** |
|  | large animal*.ti,ab,kw,hw. | ***Embase*** |
| **7** | **or/1-6 [LARGE ANIMAL MODELS]** | ***Pubmed (Medline)*** |
|  | **or/1-6** | ***Embase*** |
| **8** | (Ross adj4 (procedure* or operat* or surg* or transplant* or graft* or autograft* or homograft* or heterograft* or allograft* or xenograft*)).tw,kf. | ***Pubmed (Medline)*** |
|  | (Ross adj4 (procedure* or operat* or surg* or transplant* or graft* or autograft* or homograft* or heterograft* or allograft* or xenograft*)).ti,ab,kw,hw. | ***Embase*** |
| **9** | (pulmon* adj2 (valve or valved) adj2 (conduit* or hemiconduit*)).tw,kf. | ***Pubmed (Medline)*** |
|  | (pulmon* adj2 (valve or valved) adj2 (conduit* or hemiconduit*)).ti,ab,kw,hw. | ***Embase*** |
| **10** | PPVR.tw,kf. [percutaneous pulmonary valve replacement] | ***Pubmed (Medline)*** |
|  | PPVR.ti,ab,kw,hw. | ***Embase*** |
| **11** | **or/8-10 [ PVI I ]** | ***Pubmed (Medline)*** |
|  | **or/8-10** | ***Embase*** |
| **12** | pulmonary valve/ | ***Pubmed (Medline)*** |
|  | Pulmonary valve/ | ***Embase*** |
| **13** | ((pulmon* or neopulm*) adj6 (valve or valves or valved or valvular or valvar or supravalv* or subvalv* or leaflet* or bileaflet* or cusp*)).tw,kf. | ***Pubmed (Medline)*** |
|  | ((pulmon* or neopulm*) adj6 (valve or valves or valved or valvular or valvar or supravalv* or subvalv* or leaflet* or bileaflet* or cusp*)).ti,ab,kw,hw. | ***Embase*** |
| **14** | **12 or 13 [PV (pulmonary valves) 1 ]** | ***Pubmed (Medline)*** |
|  | **12 or 13** | ***Embase*** |
| **15** | pulmonary valve insufficiency/ or pulmonary valve stenosis/ or pulmonary subvalvular stenosis/ or pulmonary atresia/ | ***Pubmed (Medline)*** |
|  | pulmonary valve insufficiency/ or pulmonary valve stenosis/ or pulmonary subvalvular stenosis/ or pulmonary valve atresia/ | ***Embase*** |
| **16** | pulmonary artery/ or pulmonary circulation/ | ***Pubmed (Medline)*** |
|  | exp pulmonary artery / or lung circulation/ | ***Embase*** |
| **17** | ((pulmon* or neopulm* or cavopulm*) adj3 (arter* or conduit* or hemiconduit* or circulat* or root* or trunk*)).tw,kf. | ***Pubmed (Medline)*** |
|  | ((pulmon* or neopulm* or cavopulm*) adj3 (arter* or conduit* or hemiconduit* or circulat* or root* or trunk*)).ti,ab,kw,hw. | ***Embase*** |
| **18** | ((pulmon* or neopulmon*) adj6 (transpant* or implant* or explant* or engraft* or graft* or autograft* or homograft* or isograft* or heterograft* or allograft* or xenograft*)).tw,kf. | ***Pubmed (Medline)*** |
|  | ((pulmon* or neopulmon*) adj6 (transpant* or implant* or explant* or engraft* or graft* or autograft* or homograft* or isograft* or heterograft* or allograft* or xenograft*)).ti,ab,kw,hw. | ***Embase*** |
| **19** | (pulmon* adj3 atresi*).tw,kf. | ***Pubmed (Medline)*** |
|  | (pulmon* adj3 atresi*).ti,ab,kw,hw. | ***Embase*** |
| **20** | (RPVC or RPVCs or CPA or CPAs or SPVC or SPVCs).tw,kf. | ***Pubmed (Medline)*** |
|  | (RPVC or RPVCs or CPA or CPAs or SPVC or SPVCs).ti,ab,kw,hw. | ***Embase*** |
| **21** | ((RV or (right adj3 (ventric* or chamber*))) adj6 (outflow* or OT or PA or PAs or pulmon* arter* or apex)).tw,kf. | ***Pubmed (Medline)*** |
|  | ((RV or (right adj3 (ventric* or chamber*))) adj6 (outflow* or OT or PA or PAs or pulmon* arter* or apex)).ti,ab,kw,hw. | ***Embase*** |
| **22** | (RVOT or RVOTR).tw,kf. | ***Pubmed (Medline)*** |
|  | (RVOT or RVOTR).ti,ab,kw,hw. | ***Embase*** |
| **23** | **or/15-22 [PULMONARY & RVOT ]** | ***Pubmed (Medline)*** |
|  | **or/15-22** | ***Embase*** |
| **24** | heart valves | ***Pubmed (Medline)*** |
|  | heart valve/ or pulmonary valve/ | ***Embase*** |
| **25** | heart valve prosthesis/ or heart valve prosthesis implantation/ | ***Pubmed (Medline)*** |
|  | heeart valve prosthesis/ or pulmonary valve prosthesis/ or heart valve replacement/ or pulmonary valve replacement/ or Ross | ***Embase*** |
| **26** | heart valve diseases/ or heart valve prolapse/ | ***Pubmed (Medline)*** |
|  | valvular heart disease/  or heart valve prolapse/ | ***Embase*** |
| **27** | "journal of heart valve disease".jn. | ***Pubmed (Medline)*** |
|  | "journal of heart valve disease".jn. | ***Embase*** |
| **28** | (valve or valves or valved or valvular or valvar or univalv* or monovalv* or bivalve* or trivalv* or polyvalv* or supravalv* or subvalv* or transvalv* or cusp* or unicusp* or monocusp* or leaflet* or unileaflet* or monoleaflet* or bileaflet* or trileaflet* or semilunar or biovalv* or PHV or PHVs or Gore-Tex ring*).tw,kf. | ***Pubmed (Medline)*** |
|  | (valve or valves or valved or valvular or valvar or univalv* or monovalv* or bivalve* or trivalv* or polyvalv* or supravalv* or subvalv* or transvalv* or cusp* or unicusp* or monocusp* or leaflet* or unileaflet* or monoleaflet* or bileaflet* or trileaflet* or semilunar or biovalv* or PHV or PHVs or Gore-Tex ring*).ti,ab,kw,hw. | ***Embase*** |
| **29** | (Carbomedics or tilting-disc* or Medtronic Hall or Bjork-Shiley or Omniscience or cage-ball or Starr-Edwards or Medtronic Mosaic or Carpentier Edwards or Delrin or Freestyle or Edwards Prima or Prima Plus or Hancock or Pericarbon or Perimount or pericardial xenograft* or pericardial patch graft* or Labcor or SynerGraft or Contegra or (bovine adj2 jugular adj2 (vein or venous) adj4 (conduit* or graft*)) or BJVC or BJVCs).tw,kf. | ***Pubmed (Medline)*** |
|  | (Carbomedics or tilting-disc* or Medtronic Hall or Bjork-Shiley or Omniscience or cage-ball or Starr-Edwards or Medtronic Mosaic or Carpentier Edwards or Delrin or Freestyle or Edwards Prima or Prima Plus or Hancock or Pericarbon or Perimount or pericardial xenograft* or pericardial patch graft* or Labcor or SynerGraft or Contegra or (bovine adj2 jugular adj2 (vein or venous) adj4 (conduit* or graft*)) or BJVC or BJVCs).ti,ab,kw,hw. | ***Embase*** |
| **30** | **or/24-29 [VALVES]** | ***Pubmed (Medline)*** |
|  | **or/24-29** | ***Embase*** |
| **31** | **23 and 30 [PV 2]** | ***Pubmed (Medline)*** |
|  | **23 and 30** | ***Embase*** |
| **32** | **14 or 31 [PV 1 + 2]** | ***Pubmed (Medline)*** |
|  | **14 OR 31** | ***Embase*** |
| **33** | prosthesis implantation/ or blood vessel prosthesis implantation/ or heart valve prosthesis implantation/ | ***Pubmed (Medline)*** |
|  | prosthesis implantation/ or blood vessel implantation/ or heart valve replacement/ or pulmonary valve replacement/ or Ross procedure/ | ***Embase*** |
| **34** | transplantation/ or tissue transplantation/ or transplantation, autologous/ or transplantation, heterologous/ or transplantation, heterotopic/ or exp transplantation, homologous/ | ***Pubmed (Medline)*** |
|  | Transplantation/ or exp allograft/ or exp autograft/ or orthotopic transplantation/ or tissue transplantation/ or autotransplantation/ or xenograft/ or heterotopic transplantation/ or allotransplantation/ | ***Embase*** |
| **35** | exp transplants/ | ***Pubmed (Medline)*** |
|  | Geen aparte subject heading voor *exp transplants/* | ***Embase*** |
| **36** | exp host vs graft reaction/ or graft enhancement, immunologic/ or graft occlusion, vascular/ | ***Pubmed (Medline)*** |
|  | exp graft versus host reaction/ or graft occlusion/ |  |
|  | geen aparte subject heading voor *graft enhancement, immunologic/* | ***Embase*** |
| **37** | tr.fs. | ***Pubmed (Medline)*** |
|  | No equivalent | ***Embase*** |
| **38** | (implant* or preimplant* or explant* or transplant* or autotranspl* or homotranspl* or isotranspl* or heterotranspl* or allotranspl* or xenotranspl* or engraft* or graft* or autograft* or homograft* or isograft* or heterograft* or allograft* or xenograft*).tw,kf. | ***Pubmed (Medline)*** |
|  | (implant* or preimplant* or explant* or transplant* or autotranspl* or homotranspl* or isotranspl* or heterotranspl* or allotranspl* or xenotranspl* or engraft* or graft* or autograft* or homograft* or isograft* or heterograft* or allograft* or xenograft*).ti,ab,kw,hw. | ***Embase*** |
| **39** | (valv* adj3 insert*).tw,kf. | ***Pubmed (Medline)*** |
|  | (valv* adj3 insert*).ti,ab,kw,hw. | ***Embase*** |
| **40** | (((RVOT or (right adj2 (ventric* or chamber* or heart)) or ((pulmon* or neopulm*) adj3 (valv* or trunk or root)) or PV or conduit*) adj5 reconstruct*) or RVOTR).tw,kf. | ***Pubmed (Medline)*** |
|  | (((RVOT or (right adj2 (ventric* or chamber* or heart)) or ((pulmon* or neopulm*) adj3 (valv* or trunk or root)) or PV or conduit*) adj5 reconstruct*) or RVOTR).ti,ab,kw,hw. | ***Embase*** |
| **41** | reconstruct*.tw,kf. and pulm* arter*.mp. | ***Pubmed (Medline)*** |
|  | reconstruct*.ti,ab,kw,hw. and pulm* arter*.mp. | ***Embase*** |
| **42** | ((RVOT or right ventricular outflow or PV or valv* or cusp* or posterior leaflet* or conduit*) adj5 replac*).tw,kf. | ***Pubmed (Medline)*** |
|  | ((RVOT or right ventricular outflow or PV or valv* or cusp* or posterior leaflet* or conduit*) adj5 replac*).ti,ab,kw,hw. | ***Embase*** |
| **43** | **or/33-42 [IMPLANTATION ; GRAFT]** | ***Pubmed (Medline)*** |
|  | **Or/33-42** | ***Embase*** |
| **44** | **32 and 43 [PVI II ]** | ***Pubmed (Medline)*** |
|  | **32 and 43** | ***Embase*** |
| **45** | **11 or 44 [ PVI I + II ]** | ***Pubmed (Medline)*** |
|  | **11 or 44** | ***Embase*** |
| **46** | **45 and 7 [ LARGE ANIMAL PVI ]** | ***Pubmed (Medline)*** |
|  | **45 and 7** | ***Embase*** |
| **47** | **remove duplicates from 46** |  |
