# Supplementary material for: Pulmonary valve tissue engineering strategies in large animal models
Source: PLoS One. 2021 Oct 5;16(10):e0258046. doi: 10.1371/journal.pone.0258046 (PMC8491907; doi:10.1371/journal.pone.0258046)
Supplement: S2 Table — A. Questionnaire of the quality assessment. B. Questions on the control groups. (DOCX) [file pone.0258046.s004.docx]

**S2-A Table. Questionnaire of the quality assessment**.

|  | **ANIMAL information** |
| --- | --- |
| Q1 | Is the animal species described? |
| Q2 | Is the strain described? |
| Q3 | Is the number of animals described per experimental group? |
| Q4 | Is the sex of the animals described? |
| Q5 | Is the age or weight described? |
| Q6 | Ethical review permission described? |
|  | **STUDY design** |
| Q7 | Is the allocation of animals to experimental group (follow-up time) clear? |
| Q8 | Is the duration of the follow-up time of the explants clear? |
| Q9 | Was attendance of control group clear described? ****see S2b table*** |
| Q10 | Was random allocation to the groups clear described? |
| Q11 | Was the qualitatively echo assessment performed in blinded fashion? |
| ***note*** | This is applicable if echo analyses which was qualitatively evaluated (Valve mobility score, regurgitation score, quality). In case the study conducted only quantitative echo analyses, this question was not applicable. |
|  | **ADVERSE events** |
| Q12 | Are adverse events clearly stated? |
| Q13 | Are the numbers of drop-outs clearly described? |
| Q14 | Is the timepoint of the drop-outs clearly described? |
| Q15 | Are the reasons for drop-outs clearly described? |
|  | **PROCEDURE items** |
| Q16 | Is the surgical procedure clearly described? |
| Q17 | Is the diameter of the implanted valve described? |
| Q18 | Is the composition of the heart valve scaffold clearly described? |
|  | **TISSUE ENGINEERING items** |
| Q19 | Sterilisation/desinfection clearly described? |
| Q20 | Banking prior to implantation clearly described? |
| Q21 | The (active) pre-seeding/pre-treatment procedure clearly described? |
| Q22 | Is the decellularization procedure clearly described? |

**S2-B Table. Questions on the control groups.**

| Additional question Q9 | |
| --- | --- |
|  | Is there a control group |
|  | ***in case Yes:*** |
|  | *Yes, type A (non-operated sheep or sham) for echo analyses ((only)* |
|  | *Yes, type B. positive or negative control animal for echo analyses (only)* |
|  | *Yes, type C comparative control group (bioprostheses) (only)* |
|  | *Yes, multiple types of control (A, B and or C)* |
|  | ***in case no:*** |
|  | *No; Not specified by the ref. authors* |
|  | *No; but specified and described by the ref authors* |
|  | ***in case no and not described by the authors, what could be a possible reason underneath?*** |
|  | *No; Possible reason: Study seems like a pilot or feasibility study.* |
| ***note*** | Yes, in case the word 'feasibility' or 'pilot' or equivalent was mentioned in the manuscript. Or in case an acute (hours) duration of the follow-up. |
|  | *No; One TE scaffold analyzed (e.g., only differ in FU-time)* |
