# Supplementary material for: Pulmonary valve tissue engineering strategies in large animal models
Source: PLoS One. 2021 Oct 5;16(10):e0258046. doi: 10.1371/journal.pone.0258046 (PMC8491907; doi:10.1371/journal.pone.0258046)
Supplement: S3 Table — (DOCX) [file pone.0258046.s005.docx]

**S3 Table. Reported anticoagulation therapies.**

| **NATURAL SCAFFOLDS** | **Author [REF]** | **Anticoagulation therapy** | **Dose** |
| --- | --- | --- | --- |
|  | Helder [89] | Heparin | 25 U/kg 2dd for 1 week |
|  | Vincentelli [71] | Aspirin | 500 mg/day for 1 week |
|  | Navarro [90] | Aspirin and dipyridamole | 100 mg/day and 75mg/d till explant |
|  | Goecke [82] | Dalteparin | 5000 IE/day for 2 weeks |
|  | van Steenberghe [97] | LMWH | 40 mg/day for 5 days |
|  | Gallo 2x [80 & 81] | Lysine acetylsalicylate | 100 mg/day for 15 days |
|  | Knirsch [102] | Acetysalicylic acid | 100 mg/day for 5 days |
|  | Hennessy [104] | Warfarin | Dosis unknow; 2 days |
|  |  |  |  |
| **SYNTHETIC SCAFFOLDS** | Dijkman [29] | Acetylsalicylic acid | Dosis unknow; until termination |
|  | Spriestersbach [53] | Acetylsalicylic acid + dalteparin | 100 mg/day 5 days |
|  | Emmert [50] | Acetylsalicylic acid | 100 mg/day 5 days |
|  | Kluin [15] | Ascal | 80 mg/daily until termination |
|  | Schmitt [105] | Acetylsalicylic acid | 100 mg/day 5 days |
|  | Miller [45] | Aspirin | 5 mg/day until termination |
|  | Weber [40 and 54] | Aspirin + warfarin | Dosis unknow; until termination |
|  | Soliman [47] | Enoxaparin and Aspirin | 20 mg 2dd and250 mg 1dd for 5 days |
|  | Reimer [52] | Heparin | 750 IU/daily until termination |
|  | Syedain [38] | Heparin | 2000 IE until termination |
|  | Yamanami [41] | LMWH | 50 U/kg/day for 1 week |
|  | Driessen-Mol [49] | Yes, but not specified | - |
|  | Rijswijk [99] | Ascal | Lifelong |
|  | Schmidt [34] | Yes, but not specified | - |
