# Supplementary material for: Pulmonary valve tissue engineering strategies in large animal models
Source: PLoS One. 2021 Oct 5;16(10):e0258046. doi: 10.1371/journal.pone.0258046 (PMC8491907; doi:10.1371/journal.pone.0258046)
Supplement: S4 Table — A. Study characteristics of the pre-clinical studies on synthetic scaffolds. The applied strategy number correlates with the numbers in Fig 4. NR; Not reported. Ns: Not specified. PV; Pulmonary valve. AV: Aorta valve. PVR: Pulmonary valve replacement. AV: Aorta valve. BU; Bis-urea ECM; Extracellular matrix P(L,DL)LA; Poly(L-lactide-co-D,L-lactide). P4HB; Poly-4-hydroxybutyrate PC; Poly-carbonate PCL; Polycaprolactone PCUU; Poly-carbonate urethene urea PDO; Poly(1,4-dioxan-2-one) PGA; Polyglycolic Acid. PHO; Polyhydroxyoctanoate PV; Pulmonary valve. SIS; Small intestinal submucosa TE; Tissue engineering TEHV; Tissue engineered heart valve. UPy; 2-ureido-4[1H]-pyrimidinone. B. Study characteristics of the pre-clinical studies on natural scaffolds. AV; Aorta valve, F; Female, M; Male, Ns: Not specified, PV; Pulmonary valve, PVR: Pulmonary valve replacement, RVOT; Right ventricle outflow trac,. SIS-ECM; Small intestinal submucosa-extra cellular matrix, TE; Tissue engineering. (DOCX) [file pone.0258046.s006.docx]

**S4-A Table. Study characteristics of the pre-clinical studies on synthetic scaffolds.**

| **Author [REF]** | **Year of publication** | **no. of animals included** | **Scheduled Follow-up time (months)** | **Animal Species (strain) and age.** | **Animal gender** | **Implantation** | **Scaffold** | **Classification Scaffold** |
| --- | --- | --- | --- | --- | --- | --- | --- | --- |
| **Dijkman [29]** | 2012 | 1 | 1,84 | Sheep (ns); adult(ns) | NR | PVR, open surgery | PGA/P4HB | 5; *in vitro* TE (s) |
|  |  | 1 | 0,92 |  |  |  |  |  |
|  |  | 1 | NR |  |  |  |  |  |
|  |  | 2 | 0,92 |  |  |  |  |  |
| **Flanagan [30]** | 2009 | 3 | 3,00 | Sheep(s); adult (ns) | F | PV, intraluminal position, PV left intact, open surgery | Lyophilized ovine fibrinogen (plasminogen-free) | 2; in vitro TE (in hydrogel) |
|  |  | 3 |  |  |  | PV interposed position, PV left intact, open surgery | Lyophilized ovine fibrinogen (plasminogen-free) |  |
| **Gottlieb [31]** | 2010 | 7 | NR | Sheep (Dorset); NR | F | RVOT, open surgery | PGA/PLLA (50/50) | 5; *in vitro* TE (s) |
|  |  | 3 | NR |  |  |  |  |  |
|  |  | 3 | 1,38 |  |  |  |  |  |
|  |  | 3 | 2,30 |  |  |  |  |  |
|  |  | 3 | 4,60 |  |  |  |  |  |
| **Hoerstrup [32]** | 2000 | 1 | 1 day | Sheep (ns); lamb (ns) | NR | PVR, open surgery | PGA/P4HB | 5; *in vitro* TE (s) |
|  |  | 1 | 0,92 |  |  |  |  |  |
|  |  | 1 | 1,38 |  |  |  |  |  |
|  |  | 1 | 1,84 |  |  |  |  |  |
|  |  | 1 | 3,68 |  |  |  |  |  |
|  |  | 1 | 4,60 |  |  |  |  |  |
| **Kalfa [33]** | 2010 | 2 | 1,00 | Sheep (ns); 3 months | NR | Monocusp Patch in the annular region, native valve intact, open surgery | PDO | 5; *in vitro* TE (s) |
|  |  | 2 | 4,00 |  |  |  |  |  |
|  |  | 2 | 8,00 |  |  |  |  |  |
|  |  | 1 | 1,00 |  |  |  |  | 3; in situ TE (s) |
|  |  | 1 | 4,00 |  |  |  |  |  |
| **Schmidt [34]** | 2010 | 3 | 0,92 | Sheep (ns); NR | NR | PV, minimal invasive, mini thoracotomy (crimped), anterolateral-thoracic access; native valve overstented | PGA/P4HB | 5; in vitro TE (s) |
|  |  | 3 | 1,61-1,84 |  |  |  | PGA/P4HB |  |
|  |  | 2 | 0,92 |  |  |  | P(L,DL)LA |  |
| **Sodian [35]** | 2000 | 1 | 0,25 | Sheep (ns) lambs (ns) | NR | RVOT, open surgery | PHO | 5; *in vitro* TE (s) |
|  |  | 1 | 1,25 |  |  |  |  |  |
|  |  | 1 | 3,25 |  |  |  |  |  |
|  |  | 1 | 4,25 |  |  |  |  |  |
|  |  | 1 | 1,25 |  |  |  |  | 3; in situ TE (s) |
| **Stock [36]** | 2000 | 1 | 0,23 | Sheep (ns); NR | NR | RVOT, open surgery | PHO (leaflets), PGA/PHO (conduit) | 5; *in vitro* TE (s) |
|  |  | 1 | 0,46 |  |  |  |  |  |
|  |  | 1 | 0,92 |  |  |  |  |  |
|  |  | 1 | 1,38 |  |  |  |  |  |
|  |  | 1 | 1,84 |  |  |  |  |  |
|  |  | 1 | 2,76 |  |  |  |  |  |
|  |  | 1 | 3,68 |  |  |  |  |  |
|  |  | 1 | 5,52 |  |  |  |  |  |
|  |  | 1 | 1,00 |  |  |  |  | 3; in situ TE (s) |
| **Sutherland [37]** | 2005 | 6 | 8,00 | Sheep (Dover/Suffolk cross); Lambs (ns) | NR | RVOT, open surgery | PGA/PLLA | 5; in vitro TE (s) |
| **Syedain [38]** | 2011 | 2 | 0,92 | Sheep(Dorset); 6 months | NR | PA, native PV either intact or made incompetent (removing 1 leaflet), open surgery | Fibrin-trombin hydrogel | 2; in vitro TE (in hydrogel) |
|  |  | 1 | 1,84 |  |  |  |  |  |
|  |  | 1 | 1,84 |  |  |  |  |  |
| **Takewa [39]** | 2018 | 9 | 1,00 | Goats (ns); NR | NR | PV, minimally invasive, transcatheter | Allogenic subcutaneous mold | 1; In body TE |
|  |  |  |  |  |  |  |  |  |
|  |  |  | 4,00 |  |  |  |  |  |
|  |  |  |  |  |  |  |  |  |
|  |  |  | 6,00 |  |  |  |  |  |
|  |  |  |  |  |  |  |  |  |
| **Weber [40]** | 2011 | 6 | 0,92 | Primates (baboon); NR | NR | PV, positioned over pv, minimally invasive, min-thoracotomy (crimped), | PGA/P4HB | 4; Cell/bioactive seeded on the fly (s) |
|  |  |  |  |  |  |  |  |  |
|  |  |  |  |  |  | PV, positioned after PV, MI, mini-thoracotomy (crimped) |  |  |
| **Yamanami [41]** | 2010 | 1 | 0,07 | Dogs(Beagle); 296.7 days | NR | RVOT, open surgery | autologous subcutaneous tissue | 1; In body TE |
|  |  | 1 | 0,69 |  |  |  |  |  |
|  |  | 1 | 2,76 |  |  |  |  |  |
| **Bennink [132]** | 2018 | 6 | 2,00 | Sheep (Swifter); 2-4 years | NR | RVOT, open surgery | PC-UPy (leaflets) (PCL-UPy for conduit) | 3; in situ TE (s) |
|  |  | 6 | 6,00 |  |  |  |  |  |
|  |  | 6 | 12,00 |  |  |  |  |  |
| **Capulli [43]** | 2017 | 4 | 15 Hours | Sheep (ns); NR | NR | PV, pv intact, minimally invasive (delivery through right ventricle, scaffold crimped). | 60/40 P4HB/gelatin, 0.2% PGA | 3; in situ TE (s) |
| **Coyan [44]** | 2019 | 1 | 1 hour | Pig (Yorkshire); NR | NR | PVR, beating heart, open surgery, | PCUU | 3; in situ TE (s) |
|  |  | 1 | 1 hour |  |  |  |  |  |
|  |  | 1 | 4 hours |  |  |  |  |  |
|  |  | 1 | 8 hours |  |  |  |  |  |
|  |  | 1 | 12 hours |  |  |  |  |  |
| **Kluin [15]** | 2017 | 1 | 2,00 | Sheep (Swifter); 2,8 years | F | PVR, open surgery | PC-BU | 3; in situ TE (s) |
|  |  | 5 | 6,00 |  |  |  |  |  |
|  |  | 4 | 12,00 |  |  |  |  |  |
| **Soliman [47]** | 2017 | 20 | 6,00 | Sheep (Swifter); 2-4 years | NR | RVOT, open surgery | "XPV", polymer unclear | 3; in situ TE (s) |
|  |  |  | 12,00 |  |  |  |  |  |
|  |  |  | 24,00 |  |  |  |  |  |
| **Driessen-Mol [49]** | 2014 | 2 | 1 day | Sheep (ns); 2,8 ± 0,1 years | NR | PV, minimal invasive, crimped, PV overstented | PGA/P4HB | 6; deNovo TEHV |
|  |  | 2 | 1,84 |  |  |  |  |  |
|  |  | 4 | 3,68 |  |  |  |  |  |
|  |  | 4 | 5,52 |  |  |  |  |  |
| **Emmert [50]** | 2018 | 1 | 12,00 | Sheep(Gray horned heathes); NR | F | PV, minimal invasive, crimped, PV over-stented | PGA/P4HB | 6; deNovo TEHV |
|  |  | 1 |  |  |  |  |  |  |
|  |  | 9 |  |  |  |  |  |  |
| **Motta [11]** | 2018 | 1 | Acute | Sheep (ns); 1 year | F | PV, minimal invasive, crimped, PV over-stented | PGA/P4HB | 6; deNovo TEHV |
|  |  | 2 | 3,68 |  |  |  |  |  |
| **Reimer [52]** | 2017 | 1 | unclear | Sheep (Dorset); 5,5±0,8 weeks | NR | RVOT, open surgery. | Fibrin Hydrogel from bovine fibrinogen (4 mg/mL), thrombin (0.38 U/mL), and Ca++ (5.0 mM) | 2; in vitro TE (in hydrogel) |
|  |  | 1 |  |  |  |  |  |  |
|  |  | 1 |  |  |  |  |  |  |
|  |  | 1 |  |  |  |  |  |  |
|  |  | 1 |  |  |  |  |  |  |
| **Schmitt [105]** | 2016 | 5 | 2,00 | Sheep (grey horned heaths); adult (ns) | NR | PV, minimal invasive, crimped, PV over-stented | PGA/P4HB | 6; deNovo TEHV |
|  |  | 5 | 4,00 |  |  |  |  |  |
|  |  | 5 | 6,00 |  |  |  |  |  |
| **Spriestersbach [53]** | 2016 | 1 | 3h | Sheep (ns); NR | F | PV, minimal invasive, crimped, PV over-stented | PGA/P4HB | 6; deNovo TEHV |
|  |  | 2 | 2,76 |  |  |  |  |  |
| **Weber [54]** | 2013 | 3 | 0,92 | Primates (chacma baboons) NR | NR | PV, minimal invasive, mini thoracotomy (crimped), anterolateral-thoracic access, pv overstented | PGA/P4HB | 6; deNovo TEHV |
|  |  | 3 | 1,84 |  |  |  |  |  |
| **Fioretta [100]** | 2020 | 2 | 4 hours | Sheep(ns); NR | NR | PVR, minimally invasive, crimped, PV over-stented | PC-BU | 4; Cell/bioactive seeded on the fly (s) |
|  |  | 2 | 0,92 |  |  |  |  |  |
|  |  | 4 | 5,52 |  |  |  |  |  |
|  |  | 1 | 4 hours |  |  |  |  | 3; in situ TE (s) |
|  |  | 2 | 0,92 |  |  |  |  |  |
|  |  | 3 | 5,52 |  |  |  |  |  |
| **Motta 101** | 2019 | 2 | 4 hours | Sheep (white alpine); NR | F | PVR, minimally invasive, crimped, PV over-stented | PGA/P4HB | 5; in vitro TE (s) |
|  |  | 1 | 4 hours |  |  |  |  |  |

**S4-A Table. Study characteristics of the pre-clinical studies on synthetic scaffolds.** The applied strategy number correlates with the numbers in figure 4. NR; Not reported. Ns: not specified. PV; Pulmonary valve. AV: aorta valve. PVR: pulmonary valve replacement. *AV: Aorta valve. BU; Bis-urea ECM; Extracellular matrix P(L,DL)LA; Poly(L-lactide-co-D,L-lactide). P4HB; Poly-4-hydroxybutyrate PC; Poly-carbonate PCL; Polycaprolactone PCUU; Poly-carbonate urethene urea PDO; Poly(1,4-dioxan-2-one) PGA; Polyglycolic Acid. PHO; Polyhydroxyoctanoate PV; Pulmonary valve. SIS; Small intestinal submucosa TE; Tissue engineering TEHV; Tissue engineered heart valve. UPy; 2-ureido-4[1H]-pyrimidinone*

**S4-B Table. Study characteristics of the pre-clinical studies on natural scaffolds.**

| **First Author[ref]** | **Publication Year** | **no. of animals included** | **Scheduled Follow-up time (months)** | **Animal Species (strain) and age.** | **Animal gender** | **Implantation** | **Scaffold** | **Classification Scaffold** |
| --- | --- | --- | --- | --- | --- | --- | --- | --- |
| **da Costa [73]** | 2004 | 4 | 2,96/4,93 (90/150 days) | Sheep (Suffolk); 3-6 months | NR | PVR, open surgery | Porcine PV | 10; in situ TE (Decel. Xeno-/autograft) |
| **Al Hussein [103]** | 2020 | 10 | 6,0 | Sheep (Tigaie metis); NR | NR | RVOT, open surgery | Ovine PV | 10; in situ TE (Decel. Xeno-/autograft) |
| **Boldt [57]** | 2013 | 5 | 3,0 | Sheep (ns); 6-9 months | NR | PV, minimally invasive, 'folded', PV over-stented | Porcine PV + ovine SIS | 7:in vitro TE (n) |
|  |  | 5 | 3,0 |  |  |  |  |  |
| **Della Barbera [74]** | 2015 | 3 | 20-21 | Sheep (ns); 6.6 +/-2.3 months | F | PVR, open surgery | Ovine PV | 10; in situ TE (Decel. Xeno-/autograft) |
|  |  | 3 | 20-21 |  |  |  |  |  |
| **Dodge-Khatami [75]** | 2012 | 1 | acute | Sheep (Gotlandsfar); lambs (ns) | NR | PV, minimally invasive, crimped, PV over-stented | Equine valved jugular vein | 10; in situ TE (Decel. Xeno-/autograft) |
|  |  | 6 | 6,0 |  |  |  |  |  |
| **Dohmen [58]** | 2003 | 1 | 0,2 | Sheep (ns); 3 months | NR | RVOT, open surgery | Porcine PV | 7; in vitro TE (n) |
|  |  | 4 | 3,0 |  |  |  |  |  |
|  |  | 3 | 6,0 |  |  |  |  |  |
| **Dohmen [ 3]** | 2006 | 3 | 3,0 | Sheep (ns)); lambs (ns) | NR | PVR, open surgery | Porcine PV | 7; In vitro TE (n) |
|  |  | 3 | 6,0 |  |  |  |  |  |
|  |  | 7 | 3,0 |  |  |  |  | 10; in situ TE (Decel. Xeno-/autograft) |
|  |  |  | 6,0 |  |  |  |  |  |
| **Dohmen [59]** | 2006 | 4 | 9±1,8 | Sheep (ns)); 12+/-1 weeks | Mixed | PVR, open surgery | Porcine PV | 10; in situ TE (Decel. Xeno-/autograft) |
| **Elkins [76]** | 2001 | 2 | 2,00 | Sheep (Suffolk); 4-6 months | F or neutered male | RVOT, open surgery | Ovine PV | 10; in situ TE (Decel. Xeno-/autograft) |
|  |  | 2 | 4,00 |  |  |  |  |  |
|  |  | 9 | NR |  |  |  | Porcine AV |  |
| **Elkins [77]** | 2001 | 2 | NR | Sheep (ns); 3-6 months | NR | Unclear | Ovine PV | 10; in situ TE (Decel. Xeno-/autograft) |
|  |  | 2 | NR |  |  |  |  |  |
| **Erdbrugger [78]** | 2006 | 4 | 3,0 | Sheep (ns); lamb (ns) |  | RVOT, open surgery | Porcine PV | 10; in situ TE (Decel. Xeno-/autograft) |
|  |  | 3 | 6,0 |  |  |  |  |  |
|  |  | 4 | 11,0 |  |  |  |  |  |
| **Flameng [60]** | 2014 | 8 | 5,0 | Sheep (Lovenaar); 246 days | F | PVR, open surgery | Ovine AV | 10; in situ TE (Decel. Xeno-/autograft) |
|  |  | 6 | 5,0 |  |  |  |  | 8; Bioactive coated/seeded on the fly (n) |
| **Furlanetto [ 79]** | 2009 | 9 | unclear | Sheep (Santa Ines subspecies); <2 weeks | M | PVR, open surgery | Porcine PV | 10; in situ TE (Decel. Xeno-/autograft) |
| **Gallo [80]** | 2016 | 6 | 15,0 | Pigs (ns); 12 months | Mixed | RVOT, open surgery | Porcine AV | 10; in situ TE (Decel. Xeno-/autograft) |
|  |  | 6 | 15,0 |  |  |  |  |  |
| **Gallo [81]** | 2012 | Unclear | 6,0 | Pigs (vietnamese); 12 months | Mixed | RVOT, open surgery | Porcine AV | 10; in situ TE (Decel. Xeno-/autograft) |
| **Goecke [82]** | 2018 | 3 | 6,0 | Sheep (ns); 6 months | F | RVOT, open surgery | Ovine PV | 10; in situ TE (Decel. Xeno-/autograft) |
|  |  | 3 | 6,0 |  |  |  | Porcine PV |  |
|  |  | 3 | 6,0 |  |  |  | Ovine PV |  |
| **Helder [89]** | 2015 | 1 | 2,0 | Sheep (ns); 3-4 months | NR | PVR, open surgery | Porcine AV | 10; in situ TE (Decel. Xeno-/autograft) |
|  |  | 2 | 2,0 |  |  |  |  |  |
| **Hennessy [104]** | 2017 | 5 | 5,0 | Sheep (ns); ±4 months | Mixed | PVR, open surgery | Porcine AV | 10; in situ TE (Decel. Xeno-/autograft) |
| **Hilbert [85]** | 2004 | 3 | 4,6 | Sheep (ns); 10 months | NR | PVR, open surgery | Ovine PV | 10; in situ TE (Decel. Xeno-/autograft) |
|  |  | 3 | 4,6 |  |  |  | Ovine AV |  |
| **Hopkins [84]** | 2013 | 3 | 2,3 | Baboon (Papio); postpubescent (ns) | M | RVOT, open surgery | Decell baboon PV | 8; Cell/bioactive seeded on the fly (n) |
|  |  | 3 | 2,3 |  |  |  | Decell Human PV |  |
|  |  | 2 | 6,0 |  |  |  | Decell Human PV |  |
| **Hopkins [83]** | 2008 | 5 | 12,0 | Sheep (ns); 163+/-27.9 days | F | RVOT, open surgery | Ovine PV | 10; in situ TE (Decel. Xeno-/autograft) |
|  |  | 5 | 12,0 |  |  |  |  |  |
| **Iwai [86]** | 2007 | 3 | 1,0 | Dogs (Mongrel), gender and age NR | NR | PVR, open surgery | Porcine AV | 10; in situ TE (Decel. Xeno-/autograft) |
|  |  | 3 | 2,0 |  |  |  |  |  |
|  |  | 2 | 6,0 |  |  |  |  |  |
| **Kim [61]** | 2006 | 1 | 0,2 | Dogs (Mongrel); NR | Male | unclear, PVR?, procedure NR | Porcine PV | 7;in vitro TE (natural) |
|  |  | 1 | 0,7 |  |  |  |  |  |
| **Kim [87]** | 2004 | 1 | 0,2 | Goats (ns); NR | NR | PVR, open surgery | Porcine PV | 10; in situ TE (Decel. Xeno-/autograft) |
|  |  | 1 | 1,0 |  |  |  |  |  |
|  |  | 2 | 3,0 |  |  |  |  |  |
|  |  | 1 | 6,0 |  |  |  |  |  |
|  |  | 1 | 12,0 |  |  |  |  |  |
| **Kim [107]** | 2012 | 1 | 19,7 | Goat (ns); NR |  | RVOT or PVR (unclear), open surgery | Porcine PV | 10; in situ TE (Decel. Xeno-/autograft) |
| **Knirsch [102]** | 2020 | 4 | acute | Sheep (Swiss Mountain), lambs (ns) | F | RVOT, open surgery | pSIS-ECM | 11; SIS-ECM |
|  |  | 19 | 24,0 |  |  |  |  |  |
| **Leyh [62]** | 2003 | 5 | 6,0 | Sheep (ns); lamb (ns) | NR | PVR, open surgery | Porcine PV | 7; In vitro TE (n) |
|  |  | 5 | 9,0 |  |  |  |  |  |
|  |  | 3 | 6,0 |  |  |  |  |  |
|  |  | 3 | 12,0 |  |  |  |  |  |
| **Leyh [63]** | 2003 | 3 | 5,5 | Sheep (ns); 10-12 weeks | NR | PVR, open surgery, heart beating | Porcine PV | 10; in situ TE (Decel. Xeno-/autograft) |
|  |  | 3 | 2,8 |  |  |  | Ovine PV |  |
| **Lichtenberg [64]** | 2006 | 4 | 1,0 | Sheep(ns); 10-12 weeks | NR | PVR, open surgery | Ovine PV | 7;in vitro TE (n) |
|  |  | 3 | 3,0 |  |  |  |  |  |
|  |  | 4 | 1,0 |  |  |  |  | 10; in situ TE (Decel. Xeno-/autograft) |
|  |  | 3 | 3,0 |  |  |  |  |  |
| **Lopes [88]** | 2009 | 5 | 9,2 | Sheep (ns); 14-18 weeks | NR | PVR, open surgery | Porcine PV | 10; in situ TE (Decel. Xeno-/autograft) |
| **Lutter [65]** | 2010 | 3 | 0,9 | Sheep (ns); juvenile(ns) | NR | PV, minimally invasive, 'folded', PV over-stented | Porcine PV + SIS | 7;in vitro TE (n) |
| **Metzner [66]** | 2010 | 9 | 0,9 | Sheep (ns); NR | NR | PV, minimally invasive, 'folded', PV over-stented | Porcine PV + SIS | 7;in vitro TE (n) |
| **Miller [45]** | 2016 | 1 | unclear | Pig (Hanford); 10-24 weeks | F | RVOT, open surgery | pSIS-ECM | 11; SIS-ECM |
|  |  | 5 | 6,0 |  |  |  |  |  |
| **Navarro [90]** | 2010 | 2 | 0,2 | Sheep (Suffolk); 4±0.5 months | NR | RVOT, open surgery | Ovine PV | 10; in situ TE (Decel. Xeno-/autograft) |
|  |  | 2 | 1,0 |  |  |  |  |  |
|  |  | 2 | 3,0 |  |  |  |  |  |
|  |  | 2 | 5,9 |  |  |  |  |  |
| **Numata [67]** | 2004 | 2 | 1,0 | Pigs (mini-pigs); NR | NR | RVOT, open surgery | Porcine PV | 7;in vitro TE (n) |
|  |  | 2 | 1,0 |  |  |  |  | 10; in situ TE (Decel. Xeno-/autograft) |
| **Ota [68]** | 2005 | 15 | 0,3 | Dogs (Beagle); NR | F | PV, in pulmonary artery, side-bit partial clamp. pv intact, | Porcine AV | 8; Cell/bioactive seeded on the fly (n) |
|  |  |  | 1,0 |  |  |  |  |  |
|  |  | 12 | 0,3 |  |  |  |  |  |
|  |  |  | 1,0 |  |  |  |  |  |
|  |  | 12 | 0,3 |  |  |  |  | 10; in situ TE (Decel. Xeno-/autograft) |
|  |  |  | 1,0 |  |  |  |  |  |
| **Paniagua Gutierrez [91]** | 2015 | 7 | 6,0 | Sheep (ns); 3-4 months | NR | RVOT, open surgery | Porcine AV | 10; in situ TE (Decel. Xeno-/autograft) |
| **Quinn [93]** | 2011 | 8 | 4,6 | Sheep (ns) 160±9 days | F | RVOT, open surgery | Ovine PV | 10; in situ TE (Decel. Xeno-/autograft) |
| **Quinn [92]** | 2012 | 4 | 4,6 | Sheep (ns); 140±9 days | F | RVOT, open surgery | Ovine PV | 8; Cell/bioactive seeded on the fly (n) |
| **Quinn [94]** | 2016 | 7 | 6,0 | Sheep(ns); lambs (ns) | F | RVOT, open surgery | Ovine PV | 8; Cell/bioactive seeded on the fly (n) |
| **Ramm [98]** | 2019 | 3 | 6,0 | Sheep (ns); NR | F | RVOT, open surgery, interposition graft | Porcine PV | 10; in situ TE (Decel. Xeno-/autograft) |
|  |  | 4 | 6,0 |  |  |  |  |  |
|  |  | 3 | 6,0 |  |  |  |  |  |
|  |  | 3 | 6,0 |  |  |  |  |  |
|  |  | 3 | 6,0 |  |  |  |  |  |
| **Rasmussen [46]** | 2019 | 1 | Acute | Pig (mixed Yorkshire and Danish); NR | F | RVOT, open surgery | SIS-ECM | 11; SIS-ECM |
| **Schlegel [95]** | 2015 | 7 | Acute | Pigs (ns); 6-9 months | NR | PV, minimally invasive, crimped | Porcine PV | 10; in situ TE (Decel. Xeno-/autograft) |
| **Stamm [56]** | 2004 | 2 | 3,0 | Sheep (ns); NR | NR | PVR | Porcine AV + P3HB/P4HB | 9; Polymer coated |
| **Steinhoff [69]** | 2000 | 2 | 0,5 | Sheep (ns); 10-12 weeks | NR | PVR, open surgery | Ovine PV | 7; in vitro TE (n) |
|  |  | 2 | 0,9 |  |  |  |  |  |
|  |  | 2 | 2,8 |  |  |  |  |  |
|  |  | 4 | 2,8 |  |  |  |  | 10; in situ TE (Decel. Xeno-/autograft) |
| **Theodoridis [70]** | 2015 | 3 | 6,0 | Sheep (ns); 6.8 ± 0.4 years | NR | PVR, open surgery | Ovine PV | 8; Cell/bioactive seeded on the fly (n) |
|  |  | 3 | 12,0 |  |  |  |  |  |
|  |  | 3 | 6,0 |  |  |  |  | 7;in vitro TE (n) |
|  |  | 3 | 12,0 |  |  |  |  |  |
|  |  | 3 | 6,0 |  |  |  |  | 10; in situ TE (Decel. Xeno-/autograft) |
|  |  | 3 | 12,0 |  |  |  |  |  |
| **van Rijswijk [99]** | 2020 | 3 | 1,0 | Sheep (swifter); 3.1± 2.3 years | F | RVOT, open surgery, interposition graft | SIS-ECM | 11; SIS-ECM |
|  |  | 3 | 3,0 |  |  |  |  |  |
|  |  | 4 | 6,0 |  |  |  |  |  |
|  |  | 3 | 3,0 |  |  |  |  |  |
|  |  | 4 | 6,0 |  |  |  |  |  |
|  |  | 3 | 1,0 | Sheep (swifter); 7.5± 2.2 months |  |  |  |  |
|  |  | 3 | 3,0 |  |  |  |  |  |
|  |  | 4 | 6,0 |  |  |  |  |  |
| **van Steenberghe 97]** | 2018 | 2 | 3,0 | Pig (Landrace); young (ns) | F | PVR, open surgery | Porcine PV | 10; in situ TE (Decel. Xeno-/autograft) |
|  |  | 1 | 1,0 |  |  |  |  |  |
| **Vincentelli [71]** | 2007 | 1 | 0,0 | Sheep (Romanov); 12 weeks | NR | PVR, open surgery | Porcine PV | 8; Cell/bioactive seeded on the fly (n) |
|  |  | 1 | 0,2 |  |  |  |  |  |
|  |  | 5 | 4,0 |  |  |  |  |  |
|  |  | 1 | 0,0 |  |  |  |  |  |
|  |  | 1 | 0,2 |  |  |  |  |  |
|  |  | 5 | 4,0 |  |  |  |  |  |
| **White [21]** | 2005 | 4 | NR | Sheep (ns); NR | NR | RVOT, open surgery | SIS-ECM | 11; SIS-ECM |
| **Wilhelmi [72]** | 2003 | 3 | 6,0 | Sheep (ns); 10-12 weeks |  | RVOT, open surgery | Porcine PV | 7;in vitro TE (n) |
|  |  | 2 | 9,0 |  |  |  |  |  |
|  |  | 3 | 12,0 |  |  |  |  |  |
|  |  | 5 | 6,0 |  |  |  |  | 10; in situ TE (Decel. Xeno-/autograft) |
|  |  | 5 | 9,0 |  |  |  |  |  |
|  |  | 2 | 12,0 |  |  |  |  |  |
| **Wilson [96]** | 1995 | 3 | 1,0 | Dogs (ns); NR | NR | Left Pulmonary artery, PV intact, beating heart | Canin PV | 10; in situ TE (Decel. Xeno-/autograft) |
|  |  | 3 | 1,0 |  |  |  | Cainin AV |  |
| **Wu [55]** | 2007 | 4 | 3,7 | Sheep (ns); 1-2 year | NR | RVOT (unclear what happens to the native valve), open surgery | Porcine AV/ PHBHHx | 9; Polymer coated |
|  |  | 3 | 3,7 |  |  |  | Porcine AV | 10; in situ TE (Decel. Xeno-/autograft) |

**S4-B Table. Study characteristics of the pre-clinical studies on natural scaffolds.** *AV; Aorta valve, F; Female, M; Male, Ns: not specified, PV; Pulmonary valve, PVR: pulmonary valve replacement, RVOT; Right ventricle outflow trac,. SIS-ECM; Small intestinal submucosa-extra cellular matrix, TE; Tissue engineering.*
