# Supplementary material for: Pulmonary valve tissue engineering strategies in large animal models
Source: PLoS One. 2021 Oct 5;16(10):e0258046. doi: 10.1371/journal.pone.0258046 (PMC8491907; doi:10.1371/journal.pone.0258046)
Supplement: S5 Table — A. Tissue engineering strategies of the studies on synthetic scaffolds. BMD; Bone marrow derived, CCN-1; Cysteine-rich angiogenic protein 61. EDTA; Ethylenediamine tetraacetic acid, EPC; Endothelial progenitor cell, FB; Fibroblasts, MNCs; Mononuclear cells, N/A; Not applicable, PD; Pulse duplicator, SMCs; Smooth muscle cells. B. Tissue engineering strategies of the studies on natural scaffolds. BMD; Bone marrow derived. CCN-1; Cysteine-rich angiogenic protein 61. EDTA; Ethylenediamine tetraacetic acid. EPC Endothelial progenitor cell; FB; Fibroblasts. MNCs; Mononuclear cells. N/A; Not applicable. PD; Pulse duplicator. SMCs; Smooth muscle cells. (DOCX) [file pone.0258046.s007.docx]

**S5-A Table. Tissue engineering strategies of the studies on synthetic scaffolds***.*

| **Author** | **Year of publication** | **no. of animals included** | **Scheduled Follow-up time (months)** | **(Cryo)preservation method/storage** | **Added cells (types)** | **Tissue/cell Conditioning** | **Coating** | **pre-implantation decellularization?** | **On the fly cellularization?** |
| --- | --- | --- | --- | --- | --- | --- | --- | --- | --- |
| **Dijkman [29]** | 2012 | 1 | 1,84 | N/A | Vascular derived MFBs | diastolic pulse duplicator for 2 weeks | P4HB | No | No |
|  |  | 1 | 0,92 |  | Vascular derived MFBs, Autologous ECs | diastolic pulse duplicator for 2 weeks before and 2 weeks after endothelial cell seeding |  |  |  |
|  |  | 1 | NR |  | Vascular derived MFBs, Autologous ECs | diastolic pulse duplicator for 2 weeks before and 2 weeks after endothelial cell seeding |  |  |  |
|  |  | 2 | 0,92 |  | Vascular derived MFBs | diastolic pulse duplicator for 4 weeks |  |  |  |
| **Flanagan [30]** | 2009 | 3 | 3,00 | N/A | Autologous vascular derived (carotid a.) SMCs and FBs; ECs on luminal surface. | Bioreactor for 25 days before and 3 days after EC seeding. | No | No | No |
|  |  | 3 |  |  |  |  |  |  |  |
| **Gottlieb [31]** | 2010 | 7 | NR | N/A | Autologous neonatal BMD MSCs | in 850-cm^2^ roller bottle for 1 month | No | No | No |
|  |  | 3 | NR |  |  |  |  |  |  |
|  |  | 3 | 1,38 |  |  |  |  |  |  |
|  |  | 3 | 2,30 |  |  |  |  |  |  |
|  |  | 3 | 4,60 |  |  |  |  |  |  |
| **Hoerstrup [32]** | 2000 | 1 | 1 day | N/A | Autologous Vascular-derived (carotid a.) MFBs and ECs | 4 days static before and 14 days in pulse duplicator bioreactor after EC seeding. | P4HB | No | No |
|  |  | 1 | 0,92 |  |  |  |  |  |  |
|  |  | 1 | 1,38 |  |  |  |  |  |  |
|  |  | 1 | 1,84 |  |  |  |  |  |  |
|  |  | 1 | 3,68 |  |  |  |  |  |  |
|  |  | 1 | 4,60 |  |  |  |  |  |  |
| **Kalfa [33]** | 2010 | 2 | 1,00 | N/A | Ovine UCB-derived MSCs | In static conditions for 6 days | Type 1 collagen solution 1 day before cell-seeding | No | No |
|  |  | 2 | 4,00 |  |  |  |  |  |  |
|  |  | 2 | 8,00 |  |  |  |  |  |  |
|  |  | 1 | 1,00 |  | non-seeded | N/A | N/A |  |  |
|  |  | 1 | 4,00 |  |  |  |  |  |  |
| **Schmidt [34]** | 2010 | 3 | 0,92 | N/A | Autologous vascular derived (jugular v.) MFBs and vascular derived (caroticd a.) ECs | pulse duplicator for 11-12 days before and 2-3 days after EC seeding (NB: 4 TEHVs were cultured for 6 more days under dynamic straining to ensure coverage of the free edges) | 1.75%P4HB, fibrin | No | No |
|  |  | 3 | 1,61-1,84 |  |  |  |  |  |  |
|  |  | 2 | 0,92 |  | BMD MFBLCs and blood derived EPCs | static for 6 days, then in pulsatile flow-bioreactor for 3 days before EPC seeding, and again static for 3 days after. | fibrin |  |  |
| **Sodian [35]** | 2000 | 1 | 0,25 | N/A | Autologous vascular-derived (carotid a.) mixed arterial cells and venous derived (jugular v.) ECs | Static for 12-16 days before and 1 day after EC seeding | No | No | No |
|  |  | 1 | 1,25 |  |  |  |  |  |  |
|  |  | 1 | 3,25 |  |  |  |  |  |  |
|  |  | 1 | 4,25 |  |  |  |  |  |  |
|  |  | 1 | 1,25 |  | non-seeded | N/A |  |  |  |
| **Stock [36]** | 2000 | 1 | 0,23 | N/A | autologous vascular (carotid a.) derived medial cells and ECs | Static 4 days before and 1 day after EC seeding | Polyvinyl alcohol (24h) after manufacturing; Laminin before seeding | No | No |
|  |  | 1 | 0,46 |  |  |  |  |  |  |
|  |  | 1 | 0,92 |  |  |  |  |  |  |
|  |  | 1 | 1,38 |  |  |  |  |  |  |
|  |  | 1 | 1,84 |  |  |  |  |  |  |
|  |  | 1 | 2,76 |  |  |  |  |  |  |
|  |  | 1 | 3,68 |  |  |  |  |  |  |
|  |  | 1 | 5,52 |  |  |  |  |  |  |
|  |  | 1 | 1,00 |  | Non-seeded | N/A | unkown |  |  |
| **Sutherland [37]** | 2005 | 6 | 8,00 | N/A | Ovine BMD MSCs | 4 weeks in roller bottle | No | No | No |
|  |  |  |  |  |  |  |  |  |  |
|  |  |  |  |  |  |  |  |  |  |
| **Syedain [38]** | 2011 | 2 | 0,92 | N/A | Neonatal human dermal FBs | 2 weeks on mold with gentle rocking + 3 weeks in cyclic stretching bioreactor | no | no | No |
|  |  | 1 | 1,84 |  |  |  |  |  |  |
|  |  | 1 | 1,84 |  |  |  |  | treated with sodium azide (0.05%), 10 mM EDTA, 10 μg/mL aprotinin, and 10 μg/mL pepstatin prior to implantation to prevent leaflet shortning |  |
| **Takewa [39]** | 2018 | 9 | 1,00 | 70% ethanol at RT. | non-seeded | No | No | No | No |
|  |  |  |  |  |  |  |  |  |  |
|  |  |  | 4,00 |  |  |  |  |  |  |
|  |  |  |  |  |  |  |  |  |  |
|  |  |  | 6,00 |  |  |  |  |  |  |
|  |  |  |  |  |  |  |  |  |  |
| **Weber [40]** | 2011 | 6 | 0,92 | N/A | primate (autologous) BMD mononuclear cells | no | P4HB; fibrin | no | yes, 10 min after seeding transferred to OR. |
|  |  |  |  |  |  |  |  |  |  |
|  |  |  |  |  |  |  |  |  |  |
| **Yamanami [41]** | 2010 | 1 | 0,07 | N/A | non-seeded | No | No | No | No |
|  |  | 1 | 0,69 |  |  |  |  |  |  |
|  |  | 1 | 2,76 |  |  |  |  |  |  |
| **Bennink [132]** | 2018 | 6 | 2,00 | N/A | non-seeded | No | No | No | No |
|  |  | 6 | 6,00 |  |  |  |  |  |  |
|  |  | 6 | 12,00 |  |  |  |  |  |  |
| **Capulli [43]** | 2017 | 4 | 15 Hours | N/A | non-seeded | No | No | no | no |
| **Coyan [44]** | 2019 | 1 | 1 hour | N/A | non-seeded | no | no | no | no |
|  |  | 1 | 1 hour |  |  |  |  |  |  |
|  |  | 1 | 4 hours |  |  |  |  |  |  |
|  |  | 1 | 8 hours |  |  |  |  |  |  |
|  |  | 1 | 12 hours |  |  |  |  |  |  |
| **Kluin [15]** | 2017 | 1 | 2,00 | stored after coating at 37*C until implantation | non-seeded | no | Bovine Fibrin | no | no |
|  |  | 5 | 6,00 |  |  |  |  |  |  |
|  |  | 4 | 12,00 |  |  |  |  |  |  |
| **Soliman [47]** | 2017 | 20 | 6,00 | N/A | non-seeded | no | no | no | no |
|  |  |  |  |  |  |  |  |  |  |
|  |  |  | 12,00 |  |  |  |  |  |  |
|  |  |  | 24,00 |  |  |  |  |  |  |
| **Driessen-Mol [49]** | 2014 | 2 | 1 day | Fresh M-199 medium at 4°C | Ovine (vascular derived) cells | 4 weeks in dynamic bioreactor | Fibrin/P4HB | TritonX100 (0,25%) SD (0,25%) EDTA (0.02% ) TrisHCL buffer (50 mM), benzonase | no |
|  |  | 2 | 1,84 |  |  |  |  |  |  |
|  |  | 4 | 3,68 |  |  |  |  |  |  |
|  |  | 4 | 5,52 |  |  |  |  |  |  |
| **Emmert [50]** | 2018 | 1 | 12,00 | at 4°C | Ovine (vascular derived) MFB | 4 weeks dynamic bioreactor | Fibrin/P4HB | TritonX100 (0,25%) SD (0,25%) EDTA (0.02% ) TrisHCL buffer(50-mM), benzonase (100U/ml), MgCl_2_ (1 mmol/l) | no |
|  |  | 1 |  |  |  |  |  |  |  |
|  |  | 9 |  |  |  |  |  |  |  |
| **Motta [11]** | 2018 | 1 | Acute | NR | Allogenic Ovine (vascular derived) FBs | 4 weeks dynamic bioreactor, with medium containing L-ascorbic acid 2-phosphate (to enhance collagen deposition) | Fibrin/P4HB | TritonX100 (0,25%) SD (0,25%) EDTA (0.02% ) TrisHCL buffer(50-mM), benzonase (100U/ml), MgCl2 (1 mmol/l) | no |
|  |  | 2 | 3,68 |  |  |  |  |  |  |
| **Reimer [52]** | 2017 | 1 | unclear | NR | Allogenic Ovine (dermal) fibroblasts (mixed into scaffold 1 mill. ODFs/mL scaffold) | 2 weeks in dynamic bioreactor | No | Triton X-100 (1%), SDS (1%), deoxyribonuclease (2U/mL) | no |
|  |  | 1 |  |  |  |  |  |  |  |
|  |  | 1 |  |  |  |  |  |  |  |
|  |  | 1 |  |  |  |  |  |  |  |
|  |  | 1 |  |  |  |  |  |  |  |
| **Schmitt [105]** | 2016 | 5 | 2,00 | at 4°C | Allogenic Ovine (vascular derived) MFB | 4 weeks dynamic bioreactor | P4HB | TritonX100 (0,25%) SD (0,25%) EDTA (0.02% ) TrisHCL buffer(50-mM), benzonase (100U/ml), MgCl2 (1 mmol/l) | no |
|  |  | 5 | 4,00 |  |  |  |  |  |  |
|  |  | 5 | 6,00 |  |  |  |  |  |  |
| **Spriestersbach [53]** | 2016 | 1 | 3h | NR | Ovine (vascular derived) FB | 4 weeks in dynamic bioreactor | P4HB, fibrin as cell carrier | TritonX100 (0,25%) SD (0,25%) EDTA (0.02% ) TrisHCL buffer(50-mM), benzonase (100U/ml), MgCl2 (1 mmol/l) | no |
|  |  | 2 | 2,76 |  |  |  |  |  |  |
| **Weber [54]** | 2013 | 3 | 0,92 | M-199 medium at 4°C | Human (vascular derived) FB | 4 weeks in dynamic bioreactor | P4HB (1%), fibrin | TritonX100 (0,25%) SD (0,25%) EDTA (0.02% ) TrisHCL buffer(50-mM), benzonase (20-100U/ml), M-199 medium (washing). | no |
|  |  | 3 | 1,84 |  |  |  |  |  |  |
| **Fioretta [100]** | 2020 | 2 | 4 hours | NR | Autologous BMD MNCs | stored in preheated MEM-alpha medium for 1 hour | heparin as cell carrier | no | yes, of added cells |
|  |  | 2 | 0,92 |  |  |  |  |  |  |
|  |  | 4 | 5,52 |  |  |  |  |  |  |
|  |  | 1 | 4 hours | NR | non-seeded | NA | no | no | no |
|  |  | 2 | 0,92 |  |  |  |  |  |  |
|  |  | 3 | 5,52 |  |  |  |  |  |  |
| **Motta [101]** | 2019 | 2 | 4 hours | NR | Neonatal human dermal FBs | cultured at 37*C in dynamic bioreactor for 4 weeks. In medium supplemented with L-ascobric acid 2-phosphate (0.25 mg/ml) and TGF-B1 (5 ng/ml) | no | TritonX100 (0,25%) SD (0,25%) EDTA (0.02% ) TrisHCL buffer(50-mM), benzonase (100U/ml), MgCl2 (1 mmol/l) | no |
|  |  | 1 | 4 hours |  |  | cultured at 37*C in dynamic bioreactor for 4 weeks. In medium supplemented with L-ascobric acid 2-phosphate (0.25 mg/ml), without TGF-B1. |  |  |  |

**S5-A Table. Tissue engineering strategies of the studies on synthetic scaffolds*.*** *BMD; Bone marrow derived, CCN-1; Cysteine-rich angiogenic protein 61. EDTA; Ethylenediamine tetraacetic acid, EPC; Endothelial progenitor cell, FB; Fibroblasts, MNCs; Mononuclear cells, N/A; Not applicable, PD; Pulse duplicator, SMCs; Smooth muscle cells.*

**S5-B Table. Tissue engineering strategies of the studies on natural scaffolds.**

| **First Author[ref]** | **no. of animals included** | **Decellularization Method** | **(Cryo)preservation method/storage** | **Added cells (types)** | **Conditioning** | **Coating (any)** |
| --- | --- | --- | --- | --- | --- | --- |
| **da Costa [73]** | 4 | Deoxycholic acid (1%) and ethanol (70%) | N/A | non-seeded | N/A | no |
| **Al Hussein [103]** | 10 | Perfusion decellularisation: 0.02 % Sodium Azide (24 hours), 0.05 M Sodium hydroxide (4H) (0.25% SDS, 0.5% TRITON X-100, 0.5% DOC, 0.2% EDTA, 50 mM TRISpH to 7.4 ±0.05), (48 h). DN-ase and RNase (48 h). 0.02% Sodium (48). | 0.2% peracetic acid (1H) | N/A | N/A | no |
| **Boldt [57]** | 5 | Trypsin (0.05%) and EDTA (0.02%) | N/A | Autologous vascular derived (carotid a.) ECs and SMCs | 3 days static while seeding, then 16 days dynamic pulsatile flow bioreactor, then re-seeding for 3 days static | no |
|  | 5 |  |  | Allogenic BMD ovine EPCs. (CD133+) |  |  |
| **Della Barbera [74]** | 3 | SD (0.5%) and SDS (0.5%) for 24 hours | in PBS for 1 day at 4*C | non-seeded | N/A | no |
|  | 3 |  |  |  |  |  |
| **Dodge-Khatami [75]** | 1 | NR | NR | non-seeded | N/A | no |
|  | 6 |  |  |  |  |  |
| **Dohmen [58]** | 1 | Chemical decellularization by deoxycholic acid (0.1%) | at 4*C for <7 days | Autologous vascular derived (jugular or saphenous v.)ECs | 7 days static | Fibronectin |
|  | 4 |  |  |  |  |  |
|  | 3 |  |  |  |  |  |
| **Dohmen [ 3]** | 3 | Deoxycholic acid (0.1%) | N/A | Autologous vascular derived (jugular v.) ECs | static until sterile | Fibronectin |
|  | 3 |  |  |  |  |  |
|  | 7 |  | N/A | non-seeded | N/A | no |
| **Dohmen [59]** | 4 | Deoxycholic acid (0.1%) | Stored in antibiotic solution. | non-seeded | N/A | no |
| **Elkins [76]** | 2 | Proprietary process (hypotinic solution, enzymatic digestion, isotonic buffer). *From Brien: ribonuclease and deoxyribonuclease* | Stored in liquid nitrogen until implantation | non-seeded | N/A | no |
|  | 2 |  |  |  |  |  |
|  | 9 |  |  |  |  |  |
| **Elkins [77]** | 2 | ribonuclease and deoxyribonuclease | freezing chamber in cryopreservation apparatus at rate of 0.01*C/min to -2*C, then 1*C/min to -80*C, then transferred to liquid nitrogen. | non-seeded | N/A | no |
|  | 2 |  |  |  |  |  |
| **Erdbrugger [78]** | 4 | Deoxycholic acid (1%) | unclear | non-seeded | N/A | no |
|  | 3 |  |  |  |  |  |
|  | 4 |  |  |  |  |  |
| **Flameng [60]** | 8 |  | N/A | non-seeded | N/A | no |
|  | 6 | SDS (0.5%) and Triton X-100 (0.5%), in 0.3% NaCl, with 0,05% NaN_3_ | N/A | non-seeded | N/A | Fibronectin (3,2 ug/cm^2^), SDF-1α (32 ug/cm^2^) |
| **Furlanetto [ 79]** | 9 | Chemical oxidation, PEG, antiinflammatory and antithrombotic agents. | 50% ethanol | non-seeded | N/A | no |
| **Gallo [80]** | 6 | TRICOL process: (osmotic shock, Triton X-100 (1%) (with EDTA (0.2%)), sodium cholate), benzonase | not cryopreserved | non-seeded | N/A | no |
|  | 6 |  | RPMI 1640 with 0.3 g/L L-Glutamine and 10% Dimethylsulfoxide and 10% or 20% human albumin. Cryocooled in liquid nitrogen computerised freezer (ICECUBE 1860) with controlled temp lowering (01*C/min until -140*C). Storage between -140*C and -185*C in liquid nitrogen. |  |  |  |
| **Gallo [81]** | Unclear | TRICOL method: protease inhibitors in Triton X-100, sodium cholate, benzonase | not mentioned | non-seeded | N/A | no |
| **Goecke [82]** | 3 | Triton X-100 (0.5%), SDS (0.5%) | Incubated in sucrose (80%), freeze-dried in temperature-controlled lyophilizer. Primary drying: slow frozen (-1*C/min) to -40*C, then increased (1*C/min) to -30*C and kept for 24 hours. Secondary drying: temp increased (0.1*C/min) to +40*C, kept for 30 min, temp decreased (-1*C/min) to 5*C, kept for 1 hour. Stored at -20*C until use (within 2 weeks). Rehidrated with distilled water. | non-seeded | N/A | no |
|  | 3 |  |  |  |  |  |
|  | 3 |  | N/A, used fresh |  |  |  |
| **Helder [89]** | 1 | SDS (1%), DNAse (2%), MgCl2 (1M), Tris buffer (1M) | Sterilisation with 1500 Gy gamma irradiation | non-seeded | N/A | no |
|  | 2 |  | Sterilisation with 3000 Gy gamma irradiation |  |  |  |
| **Hennessy [104]** | 5 | SDS (1%), DNAse (2%), diH2O, MgCL2, Tris buffer, PAA (1%) | Sterilisation with supercritical carbon dioxide. | non-seeded | N/A | no |
| **Hilbert [85]** | 3 | N-lauroyl sarcosinate, Benzonase | At 4*C, for 2 and 27 days before decell, and 17-69 days after decell. | non-seeded | N/A | no |
|  | 3 |  | At 4*C, For 19 days before decell, and 17-69 days after decell. |  |  |  |
| **Hopkins [84]** | 3 | N-lauroyl sarcosinate ant Triton-X-100, reciprocating osmolality wash solutions, bensonase and ethanol (40%) | cryopreserved, method NR | non-seeded |  | no |
|  | 3 |  | cryopreserved, method NR | non-seeded | conditioned in solutions (see coating) | hypertonic solution, followed by a solution designed to acidify and strenghten the reversible collagen hydrogen bonding, restore water soluble hydrogel ECM components, minimize collagen fraying and act as an anti-calcification agent, finally solution to restore plasma proteins. (solutions not specified) |
|  | 2 |  |  |  |  |  |
| **Hopkins [83]** | 5 | N-lauroyl sarcosinate in TRIS buffer and Bensonase | cryopreserved in RPMI + DMSO (10%) + FBS (10%) at -1*C/min, stored in liquid nitrogen for >48h, then decell, then stored at 1*C to 10*C in saline | non-seeded | N/A | no |
|  | 5 |  | cryopreserved in RPMI + DMSO (10%) + FBS (10%) at -1*C/min, stored in liquid nitrogen for >48h, then decell, then glycerolization for 24 hours and storage at -80*C. |  |  |  |
| **Iwai [86]** | 3 | SDS (0.5%) and Triton X-100 (0.5%), in 0.3% NaCl, with 0,05% NaN3 | Stored in buffered storage solution (HEPES (10 mM), EDTA (20 mM), NaCl (0.6%), and NaN3 (0.05%)) at room temp. | non-seeded | N/A | no |
|  | 3 |  |  |  |  |  |
|  | 2 |  |  |  |  |  |
| **Kim [61]** | 1 | Triton X-100 (0.5%), NH3 (0.05%) | N/A | Autologous BMD EC-like cells and MF-like cells (labeld with fluorescence) | 1 week static | no |
|  | 1 |  |  |  |  |  |
| **Kim [87]** | 1 | NaCL (1.5 mol/L), SDS (0.5%). | Frozen with 7% Dextran-6% sucrose-1mM EDTA at -70*C until use | non-seeded | N/A | no |
|  | 1 |  |  |  |  |  |
|  | 2 |  |  |  |  |  |
|  | 1 |  |  |  |  |  |
|  | 1 |  |  |  |  |  |
| **Kim [107]** | 1 | NaCL (1.5 mol/L), SDS (0.5%). | DMSO, frozen at controlled rate to -80*C | non-seeded | N/A | no |
| **Knirsch [102]** | 4 | NA | N/A | non-seeded | NA | no |
|  | 19 |  |  |  |  |  |
|  |  |  |  |  |  |  |
|  |  |  |  |  |  |  |
| **Leyh [62]** | 5 | Trypsin (0,05%) and EDTA (0,02%) | NR | Autologous vascular (carotid a.) derived MFBs and ECs | 5 days in perfusion bioreactor before and 1 day static after EC seeding | no |
|  | 5 |  |  |  |  |  |
|  | 3 |  |  | non-seeded | no |  |
|  | 3 |  |  |  |  |  |
| **Leyh [63]** | 3 | Trypsin (0.05%) and EDTA (0.02%) for 48 hours | N/A | non-seeded | N/A | no |
|  | 3 |  |  |  |  |  |
| **Lichtenberg [64]** | 4 | SD (0.5%) and SDS (0.5%) | N/A | autologous vascular derived (jugular v.) ECs | Dynamic bioreactor with pulsatile flow for unclear days | no |
|  | 3 |  |  |  |  |  |
|  | 4 |  |  | non-seeded | NA |  |
|  | 3 |  |  |  |  |  |
| **Lopes [88]** | 5 | SDS (1%), Ethanol (70%) | In Hanks solution with antibiotics at 4*C for 5-7 days before decell, and in RPMI after decell. | non-seeded | N/A | no |
| **Lutter [65]** | 3 | Trypsin (0.02%) and trypsin EDTA (0.02%) | N/A | Autologous vascular derived (carotid a.) ECs and MFBs (FBs + SMCs) | 16 days in dynamic bioreactor before and 4 days static after EC seeding. | no |
| **Metzner [66]** | 9 | Trypsin (0.05%) and EDTA (0.02%) | N/A | Autologous vascular derived (carotid a.) ECs and MFBs (FBs + SMCs) | 16 days in dynamic bioreactor before and 4 days static after EC seeding. | no |
| **Miller [45]** | 1 | Mechanical delamination of mucosa and muscularis; decellularisation with peracetic acid | N/A | non-seeded | N/A | no |
|  | 5 |  |  |  |  |  |
| **Navarro [90]** | 2 | SDS (0.1%)-based solution (PUC 1) with mechanical shaker. | In RPMI at 4*C | non-seeded | N/A | no |
|  | 2 |  |  |  |  |  |
|  | 2 |  |  |  |  |  |
|  | 2 |  |  |  |  |  |
| **Numata [67]** | 2 | Triton X (1%) and EDTA (0.02%). Rnase (20 ug/ml) and DNase (0.2 mg/ml) | 10% DMSO programmed cooled to -90*C, then stored at -196*C | Autologous vascular (femoral a.) derived ECs. | Static reseeding for 48 hours, then soaking in EB2 medium for 48 hours | no |
|  | 2 |  |  | non-seeded | No |  |
| **Ota [68]** | 15 | 0.5% sodium lauryl sulfate, 0.5% Triton-X-100, 0.3% NaCl, 0.05% NaN_3_ | N/A | non-seeded | 1 day static | Fn-HGF (1 ug/mL) |
|  |  |  |  |  |  |  |
|  | 12 |  |  |  |  | HGF (1 ug/mL) |
|  |  |  |  |  |  |  |
|  | 12 |  |  |  | N/A | no |
|  |  |  |  |  |  |  |
| **Paniagua Gutierrez [91]** | 7 | Conduit only: agarose gel (0.5%), trypsin (1,25%). Whole AV: EDTA (0.1%) and 10 KIU/ml aprotinin, hypotonic TRIS buffer (HTB; tris (10 mM), EDTA (0.1%), aprotinin (10 KIU/ml)) for 16h, SDS (0.1%) in HTB (24 hours), Rnase (1U/ml) and Dnase (50 U/ml) in TRIS-HCL (50 mM) for 4 hours, peracetic acid (0.1%) for 3h | Stored dry on moist filter paper at -40*C before decell, cryopreserved in Hanks solution (82,5 mL), HEPES buffer (2.5 mL 1 M) and DMSO (15 mL 16%), stored at -80*C after decell | non-seeded | N/A | no |
| **Quinn [93]** | 8 | N-lauroylsarcosinate and Triton-X-100, reciprocating osmolality wash solutions, bensonase and ethanol (40%) | RPMI + DMISO (10%) + FBS (10%) at -1*C/min and stored in liquid nitrogen before decell, at 4*C after decell | non-seeded | N/A | no |
| **Quinn [92]** | 4 | N-lauroyl sarcosinate and Triton-X-100, reciprocating osmolality wash solutions, bensonase and ethanol (40%) | RPMI + DMSO (10%) + FBS (10%) at -1*C/min and stored in liquid nitrogen before decell. (48 h) as well as after decell. (48 h) | non-seeded | conditioned in solutions (see coating) | hypertonic fatty alcohol with recombinant human hyaluronin solution, followed by a solution designed to acidify and strenghten the reversible collagen hydrogen bonding, restore water soluble hydrogel ECM components, minimize collagen fraying and act as an anti-calcification agent, finally solution to restore plasma proteins. (solutions not specified) |
| **Quinn [94]** | 7 | N-lauroyl sarcosinate and Triton-X-100, reciprocating osmolality wash solutions, bensonase and ethanol (40%) | Cryopreserved in RPMI-1640 with DMSO (10%) before decell, and after decell. | non-seeded | conditioned in solutions (see coating) | hypertonic fatty alcohol with recombinant human hyaluronin solution, followed by a solution designed to acidify and strenghten the reversible collagen hydrogen bonding, restore water soluble hydrogel ECM components, minimize collagen fraying and act as an anti-calcification agent, finally solution to restore plasma proteins. (solutions not specified) |
| **Ramm [98]** | 3 | 2x12h Triton X-100 (0.5%), followed by 2x12h SDS (0.5%) | at 4°C until use | non-seeded | NA | no |
|  | 4 | 2x12h Triton X-100 (0.5%), followed by 2x12h SDS (0.5%), followed by enzymatic digestion by Dnase I (150 mL of 150 U/mL) for 48h and PNGase F (2000 U/mL) for 24h. |  |  |  |  |
|  | 3 | 2x12h SDS(0.5%)/SD(0.5%) |  |  |  |  |
|  | 3 | 2x12h SDS(0.5%)/SD(0.5%), followed by enzymatic digestion by Dnase I (150 mL of 150 U/mL) for 48h and PNGase F (2000 U/mL) for 24h. |  |  |  |  |
|  | 3 | 90min Trypsin(0.125%)/EDTA(0.05%), followed by 2x12h Triton-X100 (0.5%), followed by enzymatic digestion with Dnase I (150 mL of 150 U/mL) for 48h and PNGase F (2000 U/mL) for 24h. |  |  |  |  |
| **Rasmussen [46]** | 1 | N/A | N/A | non-seeded | N/A | no |
| **Schlegel [95]** | 7 | Deoxycholic acid (1%) | N/A | non-seeded | N/A | no |
| **Stamm [56]** | 2 | Trypsin (0.05%), | N/A | non-seeded | N/A | 1% (P3HB (82%) + P4HB (18%)) |
| **Steinhoff [69]** | 2 | Tripsin (0.05%) and EDTA (0.02%) | N/A | Autologous vascular derived (carotid a.) ECs and MFBs | Static seeding of only MFBs for 6 days, then static seeding with ECs for 2 days | no |
|  | 2 |  |  |  |  |  |
|  | 2 |  |  |  |  |  |
|  | 4 |  |  | non-seeded | N/A |  |
| **Theodoridis [70]** | 3 | SD (0.5%) and SDS (0.5%) | N/A | non-seeded | N/A | CCN1-Coating (40 ug/100 ul PBS) on a rolling device for 6 hours |
|  | 3 |  |  |  |  |  |
|  | 3 |  |  | Autologous Peripheral blood MNC derived ECs | After cell seeding: Dynamic bioreactor for >10 days (the starting flow of 0.1 l/min was increased twice a day by 0.05 l/min until a final flow of 1 l/min was reached) | CCN1-Coating (40 ug/100 ul PBS) on a rolling device for 6 hours |
|  | 3 |  |  |  |  |  |
|  | 3 |  |  | non-seeded | N/A | no |
|  | 3 |  |  |  |  |  |
| **van Rijswijk [99]** | 3 | NA | N/A | Non-seeded | NA | no |
|  | 3 |  |  |  |  |  |
|  | 4 |  |  |  |  |  |
|  | 3 |  |  |  |  |  |
|  | 3 |  |  |  |  |  |
|  | 4 |  |  |  |  |  |
| **van Steenberghe 97]** | 2 | Successive baths of pure acetone, ethanol, NaOH (1N) + NaCL (7%) and H2O2 under continuous agitation | Frozen at -80*C before decell, and after decell. | non-seeded | N/A | no |
|  | 1 |  |  |  |  |  |
| **Vincentelli [71]** | 1 | hypotonic buffer (Tris (10 mmol/L), EDTA (0.1%), aprotinin (10 KIU/mL)) --> hypotonic buffer + SDS (0.1%) --> isotonic buffer (Tris (50 mmol/L), NaCL (0.15 mol/L), EDTA (0.1%), aprotinin (10 KIU/mL)). | N/A | Autologous BMD MNCs | N/A | no |
|  | 1 |  |  |  |  |  |
|  | 5 |  |  |  |  |  |
|  | 1 |  |  | Autologous BMD MSCs |  |  |
|  | 1 |  |  |  |  |  |
|  | 5 |  |  |  |  |  |
| **White [21]** | 4 | Porcine jejunum rinsed, tunica mucosa, tunica muscularis externa and tunica serosa removed by mechanical delamination, rinsed in water to remove cells, and treated with dilute peracetic acid | N/A | non-seeded | N/A | no |
| **Wilhelmi [72]** | 3 | Trypsin (0.05%) and EDTA (0.02%) | N/A | autologous vascular (jugular v.) derived FBs and ECs | 2 weeks in rotating bioreactor | no |
|  | 2 |  |  |  |  |  |
|  | 3 |  |  |  |  |  |
|  | 5 |  |  | non-seeded | N/A |  |
|  | 5 |  |  |  |  |  |
|  | 2 |  |  |  |  |  |
| **Wilson [96]** | 3 | Steps: Tris buffer + protease inhibitor (phenylmethyl-sulfonyl fluoride (0.35 ml/L)), Triton X-100 (1%), Dnase, Rnase, Triton X-100 (1%) | NR | non-seeded | N/A | no |
|  | 3 |  |  |  |  |  |
| **Wu [55]** | 4 | Trypsin (0.05%)/EDTA + Endonucleases (Dnase I (150 IU/mL) and Rnase A (100 ug/mL) | N/A | non-seeded | N/A | PHBHHx (3-5%) |
|  | 3 |  |  |  |  | No |

**S5-B Table. Tissue engineering strategies of the studies on natural scaffolds.** *BMD; Bone marrow derived. CCN-1; Cysteine-rich angiogenic protein 61. EDTA; Ethylenediamine tetraacetic acid. EPC Endothelial progenitor cell; FB; Fibroblasts. MNCs; Mononuclear cells. N/A; Not applicable. PD; Pulse duplicator. SMCs; Smooth muscle cells*
