# Supplementary material for: Pulmonary valve tissue engineering strategies in large animal models
Source: PLoS One. 2021 Oct 5;16(10):e0258046. doi: 10.1371/journal.pone.0258046 (PMC8491907; doi:10.1371/journal.pone.0258046)
Supplement: S6 Table — Results showing that study design such as inclusion of proper control groups, randomization and blinding, reporting of key items on animal characteristic such as gender and strain and needs to be improved in preclinical studies on TEHVs. (DOCX) [file pone.0258046.s008.docx]

**S6 Table. Results of quality assessment of preclinical studies.**

|  |  | **NA** | **Yes** | **No** | **Unclear** |
| --- | --- | --- | --- | --- | --- |
|  |  | n/total (%) | n/total (%) | n/total (%) | n/total (%) |
| **ANIMAL information** | |  |  |  |  |
| **1** | Is the animal species described? | 0/80 (0%) | 80/80(100%) | 0/80 (0%) | 0/80 (0%) |
| **2** | Is the strain described? | 0/80 (0%) | 43/80(54%) | 37/80(46%) | 0/80 (0%) |
| **3** | Is the number of animals described per experimental group? | 0/80 (0%) | 77/80 (96%) | 3/80(4%) | 0/80 (0%) |
| **4** | Is the sex of the animals described? | 0/80 (0%) | 33/80(41%) | 47/80(59%) | 0/80 (0%) |
| **5** | Is the age or weight described? | 0/80 (0%) | 70/80 (88%) | 10/80 (13%) | 0/80 (0%) |
| **6** | Ethical review permission described | 0/80 (0%) | 75/80(96%) | 5/80(6%) | 0/80 (0%) |
| **STUDY design** | |  |  |  |  |
| **7** | Is the allocation of animals to experimental group (follow-up time) clear? | 16/80 (20%) | 37/43(58%) | 27/43(42%) | 0/43(0%) |
| **8** | Is the duration of the follow-up time of the explants clear? | 0/80 (0%) | 77/80(96%) | 3/80(4%) | 0/80 (0%) |
| **9** | Was some sort of control group clear described? | 0/80 (0%) | 45/80(56%) | 35/80(44%) | 0/80 (0%) |
| **10** | Was random allocation to the groups clear described? | 34/80 (43%) | 5/46(11%) | 41/46(89%) | 0/46 (0%) |
| **11** | Was the qualitatively echo assessment performed in blinded fashion? | 8/80 (10%) | 2/72(3%) | 70/72(97%) | 0/72 (0%) |
| **ADVERSE events** | |  |  |  |  |
| **12** | Are adverse events clearly stated? | 2/80 (3%) | 73/78(94%) | 5/78(6%) | 0/78(0%) |
| **13** | Are the numbers of drop-outs clearly described? | 0/80 (0%) | 71/80(89%) | 9/80(11%) | 0/80(0%) |
| 14 | Is the timepoint of the drop-outs clearly described? | 41/80 (51%) | 26/39(67%) | 13/39(33%) | 0/39(0%) |
| 15 | Are the reasons for drop-outs clearly described? | 41/80 (51%) | 34/39(87%) | 5/39(13%) | 0/39 (0%) |
| **PROCEDURE items** | |  |  |  |  |
| **16** | Is the surgical procedure clearly described? | 0/80 (0%) | 74/80(93%) | 6/80(8%) | 0/80 (0%) |
| **17** | Is the diameter of the implanted valve described? | 0/80 (0%) | 54/80(68%) | 26/80(33%) | 0/80 (0%) |
| **18** | Is the composition of the heart valve scaffold clearly described? | 0/80 (0%) | 78/80(98%) | 2/80(3%) | 0/80 (0%) |
| **Continue next page >**  **TISSUE ENGINEERING items** | |  |  |  |  |
| **19** | Sterilisation/desinfection clearly described? | 1/80 (1%) | 59/79(75%) | 20/79(25%) | 0/79(0%) |
| **20** | Banking prior to implantation clearly described? | 7/80 (9%) | 51/73(70%) | 22/80(30%) | 0/73(0%) |
| **21** | The (active) pre-seeding/pre-treatment procedure clearly described? | 42/80 (53%) | 38/38(100%) | 0/38(0%) | 0/38(0%) |
| **22** | Is the decellularization procedure clearly described? | 24/80 (30%) | 54/56(96%) | 2/56(4%) | 0/56(0%) |

**S6 Table. Results of quality assessment of preclinical studies.**  Results showing that study design such as inclusion of proper control groups, randomization and blinding, reporting of key items on animal characteristic such as gender and strain and needs to be improved in preclinical studies on TEHVs.
