# Supplementary material for: Pulmonary valve tissue engineering strategies in large animal models
Source: PLoS One. 2021 Oct 5;16(10):e0258046. doi: 10.1371/journal.pone.0258046 (PMC8491907; doi:10.1371/journal.pone.0258046)
Supplement: S7 Table — (DOCX) [file pone.0258046.s009.docx]

**S7 Table. Results of reported mortality of the synthetic and natural scaffolds.**

| Author [ref] | **Total in study (n)** | **Animal species** | **Mortality (n)** | **Peri-operative (< 48 hours)** | | | **Early < 30 days** | | | **Late (> 30 days)** | | | **Endocarditis: time of dead (n)** | **Remark** |
| --- | --- | --- | --- | --- | --- | --- | --- | --- | --- | --- | --- | --- | --- | --- |
|  |  |  |  | **Structural Valve deterioration (n)** | **Other (n)** | **Details** | **Structural Valve deterioration (n)** | **other (n)** | **Details** | **Structural Valve deterioration (n)** | **other (n)** | **Details** |  |  |
| **Synthetic implanted scaffolds** | | | | | | | | | | | | | | |
| Bennink [132] | 18 | Sheep | 3 |  | 1 | arrhythmia |  | 1 | Small hole in the XPV (+ 11 days) |  |  |  | + 5 Months (n=1) |  |
| Driessen-Mol [49] | 12 | Sheep | 2 |  | 2 | Valve migration |  |  |  |  |  |  |  |  |
| Emmert [50] | 9 | Sheep | 2 |  | 1 | Valve migration |  |  |  | 1 |  | ** Severe PR At 6 months |  |  |
| Gottlieb [31] | 19 | Sheep | 9 |  | 9 | Included pancytopenia (n = 2), aortic tear (n = 2), carotid artery monitoring line complication (n= 1), central venous air (n =1), bleeding (n = 1) and agitation and hypervagal bradycardia during anesthetic emergence (n = 2) |  |  |  |  |  |  |  |  |
| Reimer [52] | 8 | Sheep | 3 | 1* | 1** | *One immobile leaflet immediately after implantation ** atrial-septal defect |  | 1 | Extensive calcific nodules along the degradable suture line. |  |  |  |  |  |
| Soliman [47] | 20 | Sheep | 3 |  | 1 | OR related |  |  |  |  |  |  | +11 days (n=1); 3 months (n=1) |  |
| Sutherland [37] | 6 | Sheep | 2 |  | 1 | Respiratoire failure |  | 1 | GI bleeding |  |  |  |  |  |
| Takewa [39] | 7 | Goats | 3 |  |  |  |  |  |  |  | 3 | *Resp failure (n=2; at 2 and 3 months); Pneumonia (n=1; 19 months) |  |  |
| Weber [40] | 6 | Primates | 1 |  | 1 | Perioperative coronary perfusion complications |  |  |  |  |  |  |  |  |
| Flanagan [30] | 5 | Sheep | 1 |  | 1 | Pericardial tamponade |  |  |  |  |  |  |  |  |
| **Natural implanted scaffolds** | | | | | | | | | | | | | | |
| Al Hussein [103] | 10 | Sheep | 1 |  |  |  |  | 1 | Cardiac tamponade (+16th) |  |  |  |  |  |
| Furlanetto [ 79] | 8 | Sheep | 1* |  |  |  |  |  |  |  |  |  |  | *cause not described |
| Knirsch [102] | 23 | Sheep | 7 |  | 5 | Bleeding (n=2), CPB weaning failed (n=2), low cardiac output (n=1) |  | 2 | Unclear dead (n=1; +1 day), cardiac tamponade (n=1; +1 day) |  |  |  |  |  |
| Miller [45] | 6 | Pig | 1 |  |  |  |  |  |  |  |  |  | +3.5 months (n=1) |  |
| Ota [68] | 39 | Dog | 5* |  |  |  |  |  |  |  |  |  |  | *Not specified. Only total n=2 infections and n=3 others |
| Ramm [98] | 16 | Sheep | 1 |  | 1 | Arrythmia |  |  |  |  |  |  |  |  |
| Wilson [96] | 6 | Dogs | 2* |  |  |  |  |  |  |  |  |  |  | *cause not described |
| Gallo [80] | 12 | Pig | 3 |  | 2 | VF (necropsy revealed native endocarditis in the tricuspid valve) (n=1); Iatrogenic complications due to AVC damage (n=1) |  |  |  |  |  |  | Unknown time point (n=1) |  |
| Gallo [81] | 17 | Pig | 6 |  | 4 | VF (n=2); arterial embolization (n=1); and oversewing non-coronary graft cusp (n=1) |  |  |  |  |  |  | +25 and +35 days (n=2) |  |
| Schlegel [95] | 7 | Pig | 1 |  | 1 | Valve migration |  |  |  |  |  |  |  |  |
| Dodge-Khatami [75] | 7 | Sheep | 4 |  | 2 | VF;Pneumothorax |  | 1 | Pneumonia |  | 1 | Bacteremia and HF |  |  |
| Dohmen [58] | 13 | Sheep | 1 |  |  |  |  |  |  |  |  |  | Unknown time point (n=1) |  |
| Dohmen [59] | 4 | Sheep | 0 |  |  |  |  |  |  |  |  |  |  |  |
| Elkins [76] | 13 | Sheep | 2 |  | 1 | Fatal arrythmia |  | 1 | RVOT trombus (+77 days) |  | 1 | None-valve related infection |  |  |
| Hopkins [83] | 10 | Sheep | 1 |  |  |  |  |  |  |  | 1 | Animal hang herself (+299 days) |  |  |
| Lichtenberg [64] | 14 | Sheep | 2 |  |  |  |  |  |  |  |  |  | +25 (n=1) & 39 days (n=1) |  |
| Lopes [88] | 5 | Sheep | 1 |  |  |  |  |  |  |  |  |  | +40 days (n=1) |  |
| Metzner [66] | 9 | Sheep | 2 |  |  |  |  |  |  |  |  |  | Unknown time point (n=2) |  |
| Navarro [90] | 8 | Sheep | 1 |  |  |  |  |  |  |  |  |  | + 30 days (n=1) |  |
| Quinn [92] | 7 | Sheep | 4 |  |  |  |  |  |  |  |  |  | Unknown time point (n=4) |  |
| Steinhoff [69] | 10 | Sheep | 1 |  |  |  |  |  |  |  |  |  | + 17 days (n=1) |  |
| van Rijswijk [99] | 20 | Sheep | 6 |  |  |  |  |  |  |  | 2 | Heart failure (days 51 and 56) | +16, 30, 60 and 70 days (n=4) |  |
| Wu [55] | 7 | Sheep | 1 |  |  |  |  |  |  |  | 1 | Thrombosis pulmonary conduit |  |  |

**S7 Table. Results of reported mortality of the synthetic and natural scaffolds** showing that the most often operation related cause of (unplanned) death was reported.
