# Supplementary material for: Pulmonary valve tissue engineering strategies in large animal models
Source: PLoS One. 2021 Oct 5;16(10):e0258046. doi: 10.1371/journal.pone.0258046 (PMC8491907; doi:10.1371/journal.pone.0258046)
Supplement: Attachment — Submitted filename: Response to the reviewer.docx [file pone.0258046.s010.docx]

PONE-D-21-04019
PULMONARY VALVE TISSUE ENGINEERING STRATEGIES IN LARGE ANIMAL MODELS
Systematic Review, Quality Assessment & Meta-Analysis
PLOS ONE

Dear Dr. Uiterwijk,

Thank you for submitting your manuscript to PLOS ONE. After careful consideration, we feel that it has merit but does not fully meet PLOS ONE’s publication criteria as it currently stands. Therefore, we invite you to submit a revised version of the manuscript that addresses the points raised during the review process.

Please submit your revised manuscript by ~~Jul 20 2021 11:59PM~~ 🡪 August 1st. If you will need more time than this to complete your revisions, please reply to this message or contact the journal office at plosone@plos.org. When you're ready to submit your revision, log on to https://www.editorialmanager.com/pone/ and select the 'Submissions Needing Revision' folder to locate your manuscript file.

Please include the following items when submitting your revised manuscript:

- A rebuttal letter that responds to each point raised by the academic editor and reviewer(s). You should upload this letter as a separate file labeled 'Response to Reviewers'.
- A marked-up copy of your manuscript that highlights changes made to the original version. You should upload this as a separate file labeled 'Revised Manuscript with Track Changes'.
- An unmarked version of your revised paper without tracked changes. You should upload this as a separate file labeled 'Manuscript'.

If you would like to make changes to your financial disclosure, please include your updated statement in your cover letter. Guidelines for resubmitting your figure files are available below the reviewer comments at the end of this letter.

If applicable, we recommend that you deposit your laboratory protocols in protocols.io to enhance the reproducibility of your results. Protocols.io assigns your protocol its own identifier (DOI) so that it can be cited independently in the future. For instructions see: <http://journals.plos.org/plosone/s/submission-guidelines#loc-laboratory-protocols>. Additionally, PLOS ONE offers an option for publishing peer-reviewed Lab Protocol articles, which describe protocols hosted on protocols.io. Read more information on sharing protocols at <https://plos.org/protocols?utm_medium=editorial-email&utm_source=authorletters&utm_campaign=protocols>.

We look forward to receiving your revised manuscript.

Kind regards,

Panayiotis Maghsoudlou
Academic Editor
PLOS ONE

Journal Requirements:

When submitting your revision, we need you to address these additional requirements.

1. Please ensure that your manuscript meets PLOS ONE's style requirements, including those for file naming. The PLOS ONE style templates can be found at

https://journals.plos.org/plosone/s/file?id=wjVg/PLOSOne_formatting_sample_main_body.pdf and

We checked all the requirements.

<https://journals.plos.org/plosone/s/file?id=ba62/PLOSOne_formatting_sample_title_authors_affiliations.pdf>

We checked all the requirements.

2. Thank you for stating the following in the Funding Section of your manuscript:

This work was funded by ZonMW, more knowledge with fewer animals (Projectnumber 114024134, 40-42600-98-433). C.R. Hooijmans is employee of SYRCLE. M. Uiterwijk is support from the Netherlands Cardiovascular Research Initiative (CVON 2012-01): The Dutch Heart Foundation, Dutch Federation of University Medical

451 Centers, the Netherlands Organization for Health Research and Development and the Royal Netherlands Academy of Sciences. D.C. van der Valk is supported by the Gravitation Program “Materials Driven Regeneration”, funded by the Netherlands Organization for Scientific Research (024.003.013).

We note that you have provided funding information that is not currently declared in your Funding Statement. However, funding information should not appear in the Acknowledgments section or other areas of your manuscript. We will only publish funding information present in the Funding Statement section of the online submission form.

Please remove any funding-related text from the manuscript and let us know how you would like to update your Funding Statement. Currently, your Funding Statement reads as follows:

This work was funded by ZonMW, more knowledge with fewer animals (Projectnumber 114024134, 40-42600-98-433). C.R. Hooijmans is employee of SYRCLE. M. Uiterwijk is support from the Netherlands Cardiovascular Research Initiative (CVON 2012-01): The Dutch Heart Foundation, Dutch Federation of University Medical Centers, the Netherlands Organization for Health Research and Development and the Royal Netherlands Academy of Sciences. D.C. van der Valk is supported by the Gravitation Program “Materials Driven Regeneration”, funded by the Netherlands Organization for Scientific Research (024.003.013).

Please include your amended statements within your cover letter; we will change the online submission form on your behalf.

We included the amended statements within our cover letter

3. In your Data Availability statement, you have not specified where the minimal data set underlying the results described in your manuscript can be found. PLOS defines a study's minimal data set as the underlying data used to reach the conclusions drawn in the manuscript and any additional data required to replicate the reported study findings in their entirety. All PLOS journals require that the minimal data set be made fully available. For more information about our data policy, please see http://journals.plos.org/plosone/s/data-availability.

Upon re-submitting your revised manuscript, please upload your study’s minimal underlying data set as either Supporting Information files or to a stable, public repository and include the relevant URLs, DOIs, or accession numbers within your revised cover letter. For a list of acceptable repositories, please see http://journals.plos.org/plosone/s/data-availability#loc-recommended-repositories. Any potentially identifying patient information must be fully anonymized.

Important: If there are ethical or legal restrictions to sharing your data publicly, please explain these restrictions in detail. Please see our guidelines for more information on what we consider unacceptable restrictions to publicly sharing data: http://journals.plos.org/plosone/s/data-availability#loc-unacceptable-data-access-restrictions. Note that it is not acceptable for the authors to be the sole named individuals responsible for ensuring data access.

We will update your Data Availability statement to reflect the information you provide in your cover letter.

4. Please include captions for your Supporting Information files at the end of your manuscript, and update any in-text citations to match accordingly. Please see our Supporting Information guidelines for more information: http://journals.plos.org/plosone/s/supporting-information.

 We included captions to the supplement figures and copied these to the end of the manuscript.

5. Please review your reference list to ensure that it is complete and correct. If you have cited papers that have been retracted, please include the rationale for doing so in the manuscript text, or remove these references and replace them with relevant current references. Any changes to the reference list should be mentioned in the rebuttal letter that accompanies your revised manuscript. If you need to cite a retracted article, indicate the article’s retracted status in the References list and also include a citation and full reference for the retraction notice.

We reviewed the reference list and updated the ISO 2015 standards to the ISO 2021 standards in the references (see also comment no. 3 of reviewer #2 (reference 116, Line 977)

[Note: HTML markup is below. Please do not edit.]

Reviewers' comments:

Reviewer's Responses to Questions

**Comments to the Author**

1. Is the manuscript technically sound, and do the data support the conclusions?

The manuscript must describe a technically sound piece of scientific research with data that supports the conclusions. Experiments must have been conducted rigorously, with appropriate controls, replication, and sample sizes. The conclusions must be drawn appropriately based on the data presented.

Reviewer #1: Yes

Reviewer #2: Yes

2. Has the statistical analysis been performed appropriately and rigorously?

Reviewer #1: Yes

Reviewer #2: Yes

3. Have the authors made all data underlying the findings in their manuscript fully available?

The [PLOS Data policy](http://www.plosone.org/static/policies.action#sharing) requires authors to make all data underlying the findings described in their manuscript fully available without restriction, with rare exception (please refer to the Data Availability Statement in the manuscript PDF file). The data should be provided as part of the manuscript or its supporting information, or deposited to a public repository. For example, in addition to summary statistics, the data points behind means, medians and variance measures should be available. If there are restrictions on publicly sharing data—e.g. participant privacy or use of data from a third party—those must be specified.

Reviewer #1: Yes

Reviewer #2: Yes

4. Is the manuscript presented in an intelligible fashion and written in standard English?

PLOS ONE does not copyedit accepted manuscripts, so the language in submitted articles must be clear, correct, and unambiguous. Any typographical or grammatical errors should be corrected at revision, so please note any specific errors here.

Reviewer #1: Yes

Reviewer #2: Yes

5. Review Comments to the Author

Please use the space provided to explain your answers to the questions above. You may also include additional comments for the author, including concerns about dual publication, research ethics, or publication ethics. (Please upload your review as an attachment if it exceeds 20,000 characters)

Reviewer #1: The authors provided a systematic review, quality assessment, and meta-analysis regarding publications on tissue engineering heart valve strategies in large animal models. This review was presented in a clear, concise, and informative way that highlights change necessary in this area of research to provide more consistent, clear, and quality translational and preclinical work. The reviewer recommends publication of this work that will likely guide better large animal study design but has a few minor comments to improve clarity of the work presented. Overall, the study design was very transparent.

Minor comments:
1. Please read through the entire text for punctuation errors (primarily with missing punctuation), correct use of plural, and tense agreement.
a. Examples: line 39 “publications”, line 140 “questions”, lines 194,195 consistency with number punctuation, 229 “subcutaneously”, line 73, 364, 376

We read the manuscript carefully and made the necessary corrections.

2. Figure 2A- caption states “shifted from mainly natural-based scaffold design before 2015, to synthetic designs after 2015,” but this wording is not clear. The majority of the studies still use natural scaffold (>50%), but the proportion is changing over time. Please be more precise with the wording.

We changed the caption of Figure 2A from:

*“Percentage of publications of studies on TEHV scaffolds shifted from mainly natural-based scaffold designs before 2015, to synthetic scaffolds designs after 2015”*

Into

*“The proportion of publications on synthetic TEHV scaffolds increased overtime”*

3. Figure 3- consider specifying what (n) and (s) mean explicitly in the figure caption.

We changed the legend item in figure 3A as follow.

Natural scaffolds

Synthetic scaffolds

From:

Natural scaffolds (n)

Synthetic scaffolds (s)

Into:

*4. Figure 4- Please reword this caption as this was extremely difficult to understand.*

We reword the caption in figure 4 as follow.

From:

**Figure 4**. **Overview of strategies in tissue engineering of heart valves.** Start with the scaffold materials (synthetic or natural), subsequent the pathways of preparation techniques prior to implantation. Finally present 11 strategies were identified*, n= numbers of valves represented in each group. TE; Tissue engineering. SIS-ECM; Small intestinal submucosa extra cellular matrix. *Decellularized. Xeno-/autografts*

Into:

**Figure 4. Overview of strategies in tissue engineering of heart valves***.* Based on all experimental groups found in literature, 11 different TE strategies were identified. Five strategies used a natural scaffold (*green; n*) and six strategies a synthetic (*orange; s*) scaffold.  *TE; Tissue engineering. SIS-ECM; Small intestinal submucosa extra cellular matrix. *Decellularized. Xeno-/autografts*

*5. Figure 6 and 7- consider adding the figure title as the y-axis label for clarity.*

We changed the figure and replaced the title to the y-axis.

*6. Discussion:
a. Of the studies where gender was reported, was the castration/intact status of the animals included? This information could be of interest in interpreting the success and/or calcification of the implanted valves.*

This is an interesting question. The castration status of the animals was not reported in any of the studies as far as the authors remember. Although some authors mentioned “fertile” status of the animals. Although, not further specified. This is an important topic because their fertility is depending on age (Age at puberty (ewe): 6 - 9 months) amongst other factors like season and the strain.

However, we did not pre-defined and did not systematic analyzed this question and so did not double checked this data. Therefor we do not want to speculate on this topic in the manuscript. Especially because the effect of castration and the hormonal effects of the animal in relation to the regeneration process is although interesting also yet unknown and complicated and reaching out of the topic of the subject of this manuscript.

*b. From the collected data, would it be possible to determine calcification status from the natural vs. synthetic valve implants in these animal models?*

This is an excellent suggestion and we are happily to announce that in the past months one of the Co-authors (D. van der Valk) used the literature data set to collect all outcomes regarding calcification and subsequently conducted a new meta-analysis. The manuscript will be submitted shortly after the current paper is accepted.

Reviewer #2: Overall an excellent contribution by Uiterwijk et al and discussion covers many important points.
Few topic if Authors can cover a bit more in details.

Thank you for these kind words.

1*: In discussion author cover about xenotransplant and how generally it can tolerate in animal model but hasn’t worked in clinic. Question is, does any of the study actually study the immune response as in evaluate both innate and adaptive immuneresponse, monitor IgG against graft, development of HLA antigen against implanted tissue. It needs to be highlighted that poorly derived conclusions with xeno animal studies can lead to major adverse events in clinic.*

The authors do agree with the reviewer that care should be taken on the translatability of results from animal derived studies on xenograft implantation. Special care needs to be taken regarding the innate but also humoral response. As the innate immuneresponse is often evaluated by (immune)histology (e.g., CD45, CD68, CD57), in lesser extent the adaptive response of T-cells and B-cells (CD8, CD3, CD11b).

The first author (M.U) scanned the publication on natural valves implanted as xenograft. This elucidated that, 28 publications of natural scaffolds implanted as xenograft described an evaluation of the innate immuneresponse and 8 studies (additionally) evaluated the adaptive immune response. Although this subject is not our expertise and not predefined in our study protocol, the authors do agree that some more attention can be made on this subject in the discussion, although with some caution.

Therefor we added the following sentence:

“Moreover, in pre-clinical studies that evaluate xenograft implantation, researchers need to take into account the adaptive immuneresponse and the model specific HLA (mis)match in their chosen donor and animal model. The authors noticed that most of the studies on natural scaffold implanted as xenografts did studied the innate immuneresponse by (immune)histology (e.g., CD45, CD68, CD57) but in lesser extent evaluated the adaptive response by T-cells, B-cells (CD8, CD3, CD11b) or immunoglobulins by panel reactive antibody test.” (Line 462-467)

*2: Great discussion on need for control. It could be expanded with more discussion if valve is for Pediatric vs. adult population and how animal age can impact outcome.*

Thank you for this suggestion. The authors do agree and added the mentioned topics for the readers when considering the use of a control valve:

“Furthermore, it should be taken into account that valve degeneration is faster in young children than in adults. The choice of the control animal must match here.” (Line 495-496)

3: ISO 5840 2021 has very clear requirement for control valve in addition to other very specific in vivo study requirement. Authors do bring the standard once, which is great. Perhaps a paragraph towards the end with bit more context from new ISO standard (issued Jan 2021) can help further standardize future animal studies.

Thank you for this suggestion. We replaced the reference to the new ISO standards (Line 356). Although the ISO standards are not developed for tissue engineered heart valves, in our opinion the standards can be can be the basis for TEHV and can be further customized with specific requirements, e.g., regarding analyzes of the recipient immune response. We added this in line 417 the following sentence:

“that can be used as basic requirements as long as no specifc ISO-standard on TEHV is available” (Line 471)

Overall, great work and really appreciate authors making an effort to put this together.

6. PLOS authors have the option to publish the peer review history of their article ([what does this mean?](https://journals.plos.org/plosone/s/editorial-and-peer-review-process#loc-peer-review-history)). If published, this will include your full peer review and any attached files.


If you choose “no”, your identity will remain anonymous but your review may still be made public.


**Do you want your identity to be public for this peer review?** For information about this choice, including consent withdrawal, please see our [Privacy Policy](https://www.plos.org/privacy-policy).

Reviewer #1: No

Reviewer #2: **Yes:** Dr. Zeeshan Syedain

[NOTE: If reviewer comments were submitted as an attachment file, they will be attached to this email and accessible via the submission site. Please log into your account, locate the manuscript record, and check for the action link "View Attachments". If this link does not appear, there are no attachment files.]

While revising your submission, please upload your figure files to the Preflight Analysis and Conversion Engine (PACE) digital diagnostic tool, <https://pacev2.apexcovantage.com/>. PACE helps ensure that figures meet PLOS requirements. To use PACE, you must first register as a user. Registration is free. Then, login and navigate to the UPLOAD tab, where you will find detailed instructions on how to use the tool. If you encounter any issues or have any questions when using PACE, please email PLOS at [figures@plos.org](mailto:figures@plos.org). Please note that Supporting Information files do not need this step.
